# Activation by oxidation and ligand exchange in a molecular manganese vanadium oxide water oxidation catalyst†

**DOI:** 10.1039/d1sc03239a

**Published:** 2021-08-30

**Authors:** Gustavo Cárdenas, Ivan Trentin, Ludwig Schwiedrzik, David Hernández-Castillo, Grace A. Lowe, Julian Kund, Christine Kranz, Sarah Klingler, Robert Stach, Boris Mizaikoff, Philipp Marquetand, Juan J. Nogueira, Carsten Streb, Leticia González

**Affiliations:** Institute of Theoretical Chemistry, University of Vienna Währinger Str. 17 1090 Vienna Austria leticia.gonzalez@univie.ac.at; Chemistry Department, Universidad Autónoma de Madrid Calle Francisco Tomás y Valiente, 7 28049 Madrid Spain; Institute of Inorganic Chemistry I, Ulm University Albert-Einstein-Allee 11 89081 Ulm Germany carsten.streb@uni-ulm.de; Institute of Analytical and Bioanalytical Chemistry, Ulm University Albert-Einstein-Allee 11 89081 Ulm Germany; Vienna Research Platform on Accelerating Reaction Discovery, University of Vienna Währinger Str. 17 1090 Vienna Austria; IADCHEM, Institute for Advanced Research in Chemistry, Universidad Autónoma de Madrid Madrid Spain

## Abstract

Despite their technological importance for water splitting, the reaction mechanisms of most water oxidation catalysts (WOCs) are poorly understood. This paper combines theoretical and experimental methods to reveal mechanistic insights into the reactivity of the highly active molecular manganese vanadium oxide WOC [Mn_4_V_4_O_17_(OAc)_3_]^3−^ in aqueous acetonitrile solutions. Using density functional theory together with electrochemistry and IR-spectroscopy, we propose a sequential three-step activation mechanism including a one-electron oxidation of the catalyst from [Mn_2_^3+^Mn_2_^4+^] to [Mn^3+^Mn_3_^4+^], acetate-to-water ligand exchange, and a second one-electron oxidation from [Mn^3+^Mn_3_^4+^] to [Mn_4_^4+^]. Analysis of several plausible ligand exchange pathways shows that nucleophilic attack of water molecules along the Jahn–Teller axis of the Mn^3+^ centers leads to significantly lower activation barriers compared with attack at Mn^4+^ centers. Deprotonation of one water ligand by the leaving acetate group leads to the formation of the activated species [Mn_4_V_4_O_17_(OAc)_2_(H_2_O)(OH)]^−^ featuring one H_2_O and one OH ligand. Redox potentials based on the computed intermediates are in excellent agreement with electrochemical measurements at various solvent compositions. This intricate interplay between redox chemistry and ligand exchange controls the formation of the catalytically active species. These results provide key reactivity information essential to further study bio-inspired molecular WOCs and solid-state manganese oxide catalysts.

## Abstract 2

Combined theoretical and experimental studies shed light on the initial steps of redox-activation of a molecular manganese vanadium oxide water oxidation catalyst.

## Graphical abstract

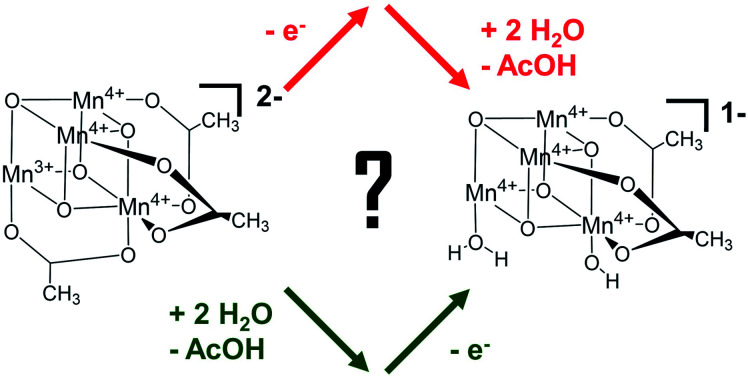

## Introduction

The development of noble metal-free water oxidation catalysts^1,2^ (WOCs) is often inspired by natural photosynthesis, where a calcium manganese oxide cubane (the oxygen evolving complex, OEC) oxidizes water near the thermodynamic potential.^3,4^ Molecular model complexes are often used in mechanistic studies of the catalytic cycle.^5–8^ Meanwhile, the design of solid-state metal oxide WOCs comes with its own set of challenges: structural complexity, typically mixed metal sites, and scarce information on the nature of their active sites.^9^ On the quest to surmount these challenges, molecular mixed metal oxides – so-called polyoxometalates (POMs) – have emerged as prototypes useful for homogeneous water oxidation catalysis and as models to correlate molecular reactivity with heterogeneous solid-state catalysts.^10^ Typically, POM-WOCs are engineered by functionalizing chemically robust POMs (mainly tungstates) with redox-active transition metals^11–13^ such as Ru,^14,15^ Co^16,17^ or Mn.^18,19^ Much research on POM-WOCs has focused on stabilizing bio-inspired polynuclear metal-oxo-aggregates [M_*x*_O_*y*_] using POM ligands, as this approach limits the oxidation state changes required per metal site during water oxidation.^11–13,20^

While the last decade has seen tremendous progress in POM-WOC synthesis and catalysis, mechanistic studies are rare, in part due to challenges related to POM-WOC stability.^21,22^ Early studies by Musaev and colleagues explored the electronic structure and accessible oxidation states of ruthenium tungstate POM-WOCs.^23^ Later, pioneering studies by Poblet and colleagues provided a water oxidation mechanism for cobalt tungstate POM-WOCs using density functional theory (DFT).^17,24^ Also, Llobet, Bo, Bonchio and colleagues combined spectroscopic and spectro-electrochemical studies with DFT calculations to investigate the redox chemistry during water oxidation by ruthenium-containing POM-WOCs.^25^ However, for many POM-WOCs^11,12,21^ mechanistic understanding of their mode of action in water oxidation catalysis is lacking.

**Fig. 1:**
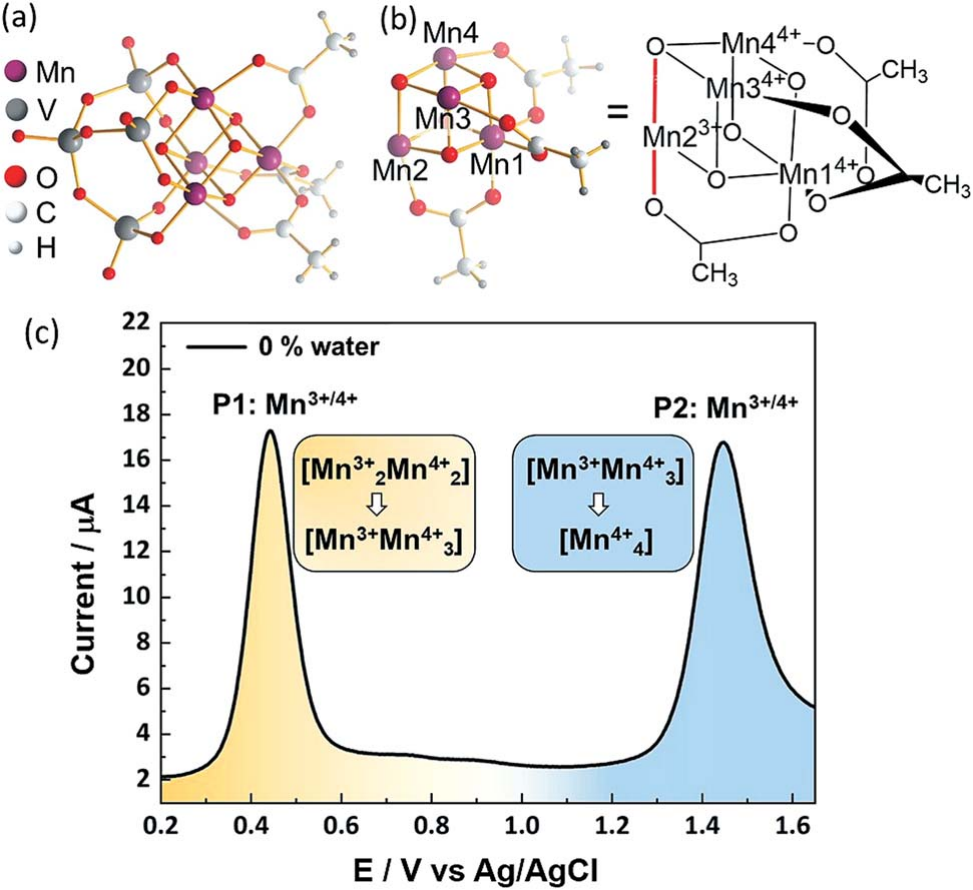
(a) Complete structure of the POM-WOC [Mn_4_V_4_O_17_(OAc)_3_]^3−^ (={Mn_4_V_4_}). (b) Simplified manganese oxo cubane core structure of the one-electron-oxidized species with Mn numbering scheme. Oxidation states of the Mn centres are indicated by superscripts. Jahn–Teller axis highlighted in red. (c) Square wave voltammogram of [Mn_4_V_4_O_17_(OAc)_3_]^3−^. Conditions: *f* = 25 Hz, *E*_SW_ = 25 mV, *E*_step_ = 2 mV water-free MeCN containing 0.1 M *n*Bu_4_NPF_6_ as electrolyte, working electrode: glassy carbon; counter-electrode Pt wire, reference electrode: Ag/AgCl (3.5 M KCl).

To date, most POM-WOC research is focused on metal-functionalized lacunary polyoxotungstates.^11–13,21^ In contrast, the use of vanadates to stabilize metal-oxo ligands is limited, possibly due to their more complex reactivity.^26^ Recently, some of us reported^19^ the embedding of OEC-inspired manganese oxo reaction centers^3^ into a molecular vanadium oxide WOC, [Mn_4_V_4_O_17_(OAc)_3_]^3−^ (={Mn_4_V_4_}), see Fig. 1. This compound is capable of electrochemical and photochemical water oxidation when operated under homogeneous conditions in aqueous acetonitrile solutions with turnover numbers > 12 000 and turnover frequencies > 200 min^−1^,^19,27^ but any knowledge related to its catalytic activity remains elusive. This insight is urgently required not only because this catalyst is one of the best-performing POM-WOCs reported, but also because it can be considered a model for the OEC and for technologically important manganese oxide water oxidation catalysts.

Herein, we report the activation mechanism of the POM-WOC [Mn_4_V_4_O_17_(OAc)_3_]^3−^ as a fundamental step towards understanding speciation in solution under catalytic conditions and elucidating the complete water oxidation cycle. Combining electrochemical measurements and theoretical calculations we unravel a three-step activation mechanism consisting of a one-electron oxidation of the catalyst, acetate-to-water ligand exchange, and a second one-electron oxidation. The calculations of the ligand exchange pathways and redox potentials allow us to precisely determine the nature of the experimentally observed redox transitions. Further, the complex interplay of proton-coupled redox processes is of general importance for understanding water oxidation catalysis and advancing the design of other efficient POM-WOCs.

## Experimental

### Electrochemistry

Voltammograms were recorded using a Gamry 1010B potentiostat and a three-electrode setup. A 3 mm diameter glassy carbon disk working electrode (CH Instruments, USA), Pt wire counter-electrode and a leakless miniature Ag/AgCl reference electrode were used. The reference electrode potential was measured against a standard calomel reference electrode (CH Instruments, USA) in pure electrolyte solution before and after each measurement. As pre-treatment, the glassy carbon (GC) electrode was cycled 10 times in 0.2 M aqueous H_2_SO_4_ between −0.5 V and 0.5 V to remove impurities. Between each square wave voltammetry (SWV) experiment, the electrode was polished with an alumina slurry. The counter-electrode Pt wire was polished with a 0.05 μm alumina slurry prior to use. Samples with varying water content were prepared by mixing the required amounts of water and acetonitrile with *n*Bu_4_NPF_6_ (0.1 M) and background SWV were collected for each solvent mixture in the absence of {Mn_4_V_4_}. Successively, (*n*Bu_4_N)_3_[Mn_4_V_4_O_17_(AcO)_3_] (32 mg, 2 mM) was added to the solution and stirred until dissolved (*ca.* 1 min).^19^ After *ca.* 5 min equilibration, the SWV was performed. Each experiment was performed at least in triplicate to ensure reproducibility. Between each experiment, the working electrode was polished to avoid interferences from possible electrode fouling (*e.g.* adsorption of intermediate species). SWV parameters were as follows: Frequency *f* = 25 Hz, pulse amplitude *E*_SW_ = 25 mV, potential step *E*_step_ = 2 mV in a range between 0.2 and 1.7 V *vs.* Ag/AgCl (3.5 M KCl) at room temperature.

### Infrared spectroscopy

IR spectra were recorded using a Fourier-transform infrared (FT-IR) spectrometer (Alpha I, Bruker Optik GmbH, Ettlingen, Germany) equipped with a zinc selenide multi-bounce attenuated total reflection (ATR) accessory (Multi ATR, Bruker Optik GmbH, Ettlingen, Germany). The spectra were collected at a spectral resolution of 4 cm^−1^ averaging 42 spectra per measurement. For each measurement, an aliquot of 0.6 ml of the sample solution (4 mM) was deposited onto the ATR crystal inside a closed sampling cell to prevent evaporation of the solvent during the measurement.

## Computational

### Ligand exchange mechanistic calculations

As in Siegbahn's studies of the OEC in photosystem II,^28,29^ calculations were carried out using the high-spin configuration and assuming that antiferromagnetic effects do not significantly affect either the reaction barriers or the geometry of the complex.^30^ Geometries were optimized using DFT and the B3LYP functional^31,32^ – as widely used in other studies of OEC model systems^33–37^ and polyoxometalate WOCs^24,38–45^—combined with the def2-SVP basis set.^46^ Local minima were confirmed by the absence of imaginary frequencies. Solvation effects were considered using the SMD implicit solvation model^47^ with a dielectric constant of 40.98. We use Grimme's D3 empirical dispersion correction^48^ and the zeroth-order DKH relativistic Hamiltonian.^49^ Electronic energies were refined using B3LYP with def2-TZVP^46^ for Mn, V, O atoms, and def2-SVP for C and H atoms. All these calculations were performed with the Gaussian 16 software package.^50^

### Redox potentials calculations

The geometries employed for the redox potential calculations were also optimized at the B3LYP/def2-SVP level of theory. Dielectric constants between 36.60 (0% water) and 38.79 (5% water) account for the variation of the solvent composition, as dictated by the experiment. The value of the dielectric constant of the mixture is calculated as:

1 *ε*_mixture_ = *φ*_water_*ε*_water_ + *φ*_acetonitrile_*ε*_acetonitrile_

where *ε*_mixture_, *ε*_water_ and *ε*_acetonitrile_ are the dielectric constants of the mixture, water (80.4) and acetonitrile (36.6), respectively, and *φ*_water_, *φ*_acetonitrile_ are the volumetric fractions of the respective solvents. Unlike the ligand exchange calculations, accurate redox potentials for OEC similar systems require the consideration of antiferromagnetic effects. This was done by employing the Orca 4.2.1 (ref. 51) suite, which enables the computation of antiferromagnetic electronic configurations using the broken-symmetry^52^ approach including implicit solvation effects. To this end, electronic energies entering the redox potentials were refined using the zeroth-order regular approximation (ZORA) for relativistic effects,^53^ along with the ZORA-Def2-SVP (C, H) and ZORA-Def2-TZVP (Mn, V, O) basis sets. The calculations used the most stable antiferromagnetic electronic configurations for the complexes in the broken-symmetry framework.^52^ Implicit solvent effects, on the single point energies, were included through the conductor-like polarizable continuum model.^54^

The standard reduction potentials 
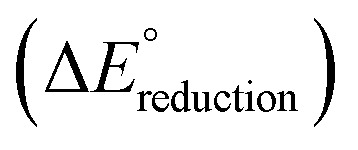
 were calculated relative to the Ag/AgCl electrode as:2
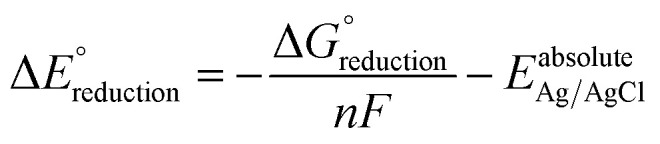
where 
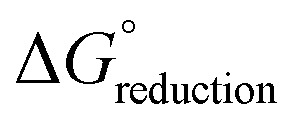
 is the change in Gibbs free energy in the reduction reaction, *n* and *F* are the number of electrons transferred and the Faraday constant, respectively, and *E*^absolute^_Ag/AgCl_ is the absolute reduction potential of the Ag/AgCl electrode. The value of *E*^absolute^_Ag/AgCl_ was obtained considering an absolute reduction potential for the Standard Hydrogen Electrode (SHE) of 4.28 V (ref. 55) and a reduction potential of 0.22 V (ref. 56) for the Ag/AgCl electrode relative to the SHE. The Gibbs free energy of reduction was computed here by the so-called direct approach,^57^ in which the solution phase reaction energy is computed as the difference between the Gibbs free energy of the product and the reactant, each obtained from an optimization-frequency calculation in a continuum solvation model. Alternatively, thermodynamic cycles can be used to compute solution phase reaction energies, as they are parametrized to obtain accurate solvation free energies.^55,57^ When computing the Gibbs free energy of solvation in a thermodynamic cycle using implicit solvation models to obtain the solution phase reaction energy, explicit vibrational corrections are not included,^58^ although there are cases in which solvation can induce changes in vibrational frequencies. Therefore, it is advisable to consider these vibrational corrections,^57,59^ as we have done here using the direct approach. We note that the direct approach has also been applied in the computation of the redox potentials of POMs analogous to the one presented in this work.^60,61^

## Results and discussion

The POM-WOC [Mn_4_V_4_O_17_(OAc)_3_]^3−^ is based on a tripodal tetravanadate ligand that coordinates to a [Mn_4_O_4_] cubane (abbreviated as [Mn_*x*_^3+^Mn_4−*x*_^4+^]); the manganese centers are further stabilized by three bridging acetate ligands, see Fig. 1a and b. Square wave voltammograms and cyclic voltammograms recorded in water-free acetonitrile show that the catalyst undergoes two quasi-reversible Mn-based oxidation reactions, [Mn_2_^3+^Mn_2_^4+^] → [Mn^3+^Mn_3_^4+^] and [Mn^3+^Mn_3_^4+^] → [Mn_4_^4+^] (labelled P1 and P2, respectively, in Fig. 1c, see also Fig. S1 and Table S1 of the ESI†).^19^ Previous studies have shown that water oxidation occurs at potentials more positive than P2, suggesting that [Mn_4_^4+^] is the species that enters the water oxidation cycle.^19^ Note that no protonation of the cluster is observed by crystallography, spectroscopy or mass spectrometry,^19^ unlike other POM-WOCs discussed in the literature.^62^

**Fig. 2:**
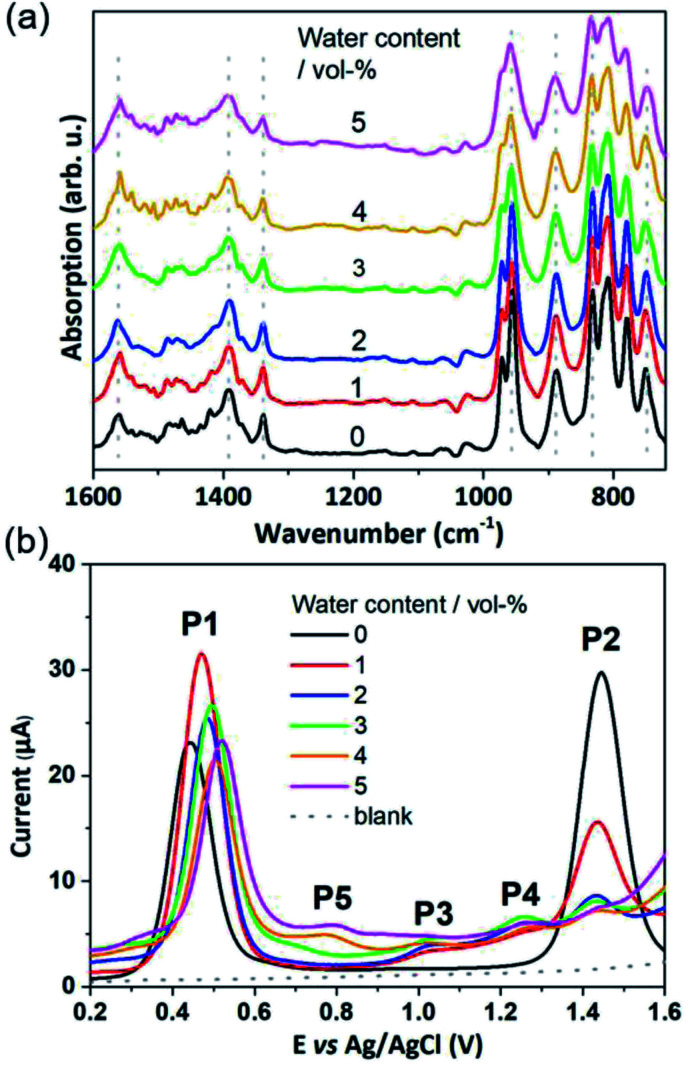
(a) IR-spectra of {Mn_4_V_4_} in acetonitrile containing 0–5 vol% of water. Each spectrum was collected after a *t* = 20 min equilibration period, which is in line with the time required for collecting the SWV data shown in (b). Note that no evidence for structural changes, ligand exchange or metal oxo framework degradation is evident. (b) Square wave voltammetry (SWV) of {Mn_4_V_4_}, MeCN : H_2_O solutions at varying water content (0–5 vol%), *f* = 25 Hz, *E*_SW_ = 25 mV, *E*_step_ = 2 mV containing 0.2 mM {Mn_4_V_4_} and 0.1 M *n*Bu_4_NPF_6_ as electrolyte.

In order to study the full catalytic cycle, first it is essential to understand how the catalyst reaches its active state. To this end, here, we investigate the P1 and P2 oxidation processes to elucidate the electronic and structural changes required to access the catalytically active species. We propose that the first step in the cluster activation is the [Mn_2_^3+^Mn_2_^4+^] → [Mn^3+^Mn_3_^4+^] oxidation (rather than ligand exchange). This is based on *in situ* IR-spectroscopic data, which was recorded at conditions identical to the experimental electrochemistry discussed below (*i.e.*, acetonitrile solutions containing up to 5 vol% water). The analysis of the characteristic metal oxide and acetate vibrations of [Mn_4_V_4_O_17_(OAc)_3_]^3−^ in the 700–1600 cm^−1^ spectral range shows no significant changes during the experimental electrochemical timescale (*i.e.*, sample preparation and experiment *approx.* 20 min; see Fig. 2a). Only the vanadate signature appears less well resolved at high water content, yet, no significant peak shift or change in intensity was observed.^63,64^ This suggests that the structural integrity of the cluster is maintained, and that no acetate-to-water ligand exchange is observed during this period. Consequently, we focus on mechanisms starting by oxidation of the native [Mn_4_V_4_O_17_(OAc)_3_]^3−^ species. Particular focus is put on exploring (i) the formation of the highly oxidized [Mn_4_^4+^] state through the [Mn^3+^Mn_3_^4+^] → [Mn_4_^4+^] redox reaction and (ii) the acetate ligand exchange reaction with water to generate the water-binding species required for subsequent O–O bond formation and oxygen evolution. Note that more complex reaction paths (including protonation or partial/full metal oxo framework hydrolysis) are principally possible but are not considered here, based on the stability studies discussed above.

To determine which redox pathways dominate at different water concentrations, the experimental redox potentials of the oxidation reaction P1 and P2 were determined at different MeCN : H_2_O ratios as a basis for subsequent theoretical calculations. To this end, we employed SWV that eliminates the capacitive current component and thus is by a few orders of magnitude more sensitive than cyclic voltammetry (CV). By varying pulse frequency and pulse amplitude and recording the forward and backward scan, information on reaction mechanism and kinetics can be obtained.^64^ In the context of this study, plotting the difference of the forward and reverse current against potential gives well-defined SWV peaks (Fig. 1c, 2b and ESI Fig. S2–S5†). These mark the experimental oxidation potentials, which can subsequently be used to compare with the calculated values derived from theory.^65^ Square wave voltammograms were recorded for the native cluster (oxidation state [Mn_2_^3+^Mn_2_^4+^]) in MeCN : H_2_O solutions (containing 0.1 M *n*Bu_4_NPF_6_ as electrolyte) with the water content varied from 0–5 vol%. Each electrochemical experiment (*i.e.*, each MeCN : H_2_O ratio) was performed using a freshly prepared catalyst solution and freshly cleaned electrodes to ensure maximum reliability and reproducibility. Each measurement was performed in triplicate. All data given are referenced against a Ag/AgCl (3.5 M) reference electrode.

In water-free, MeCN, the SWV results (Fig. 1c and 2b) correspond to the CV signals originally observed (ESI, Fig. S1†), showing an oxidative wave for the [Mn_2_^3+^Mn_2_^4+^] → [Mn^3+^Mn_3_^4+^] transition at 0.45 V (labeled P1 in Fig. 2b), and the [Mn^3+^Mn_2_^4+^] → [Mn_4_^4+^] transition at 1.45 V (P2 in Fig. 2b). When increasing the water content to 5 vol%, we observed a positive potential shift of 80 mV. In addition, the current associated with P1 initially increases when going from 0% to 1% water content. At higher water content, the P1 current decreases again as shown in Fig. 2b. With increasing water content, P2 shows a significant decrease in current, while simultaneously three new signals at less positive potentials appear. The signals are labelled in their order of appearance: P3 (1.05 V), P4 (1.25 V), and P5 (0.70 V), see Fig. 2b and ESI Fig. S2.† Initial data interpretation suggests, that the [Mn^3+^Mn_3_^4+^] → [Mn_4_^4+^] transition is strongly influenced by the presence of water, leading us to suggest that for the one-electron oxidized species [Mn^3+^Mn_3_^4+^], there may be two competing reaction pathways, *i.e.*, further oxidation (to give [Mn_4_^4+^]) or acetate-to-water ligand exchange.

**Scheme 1:**
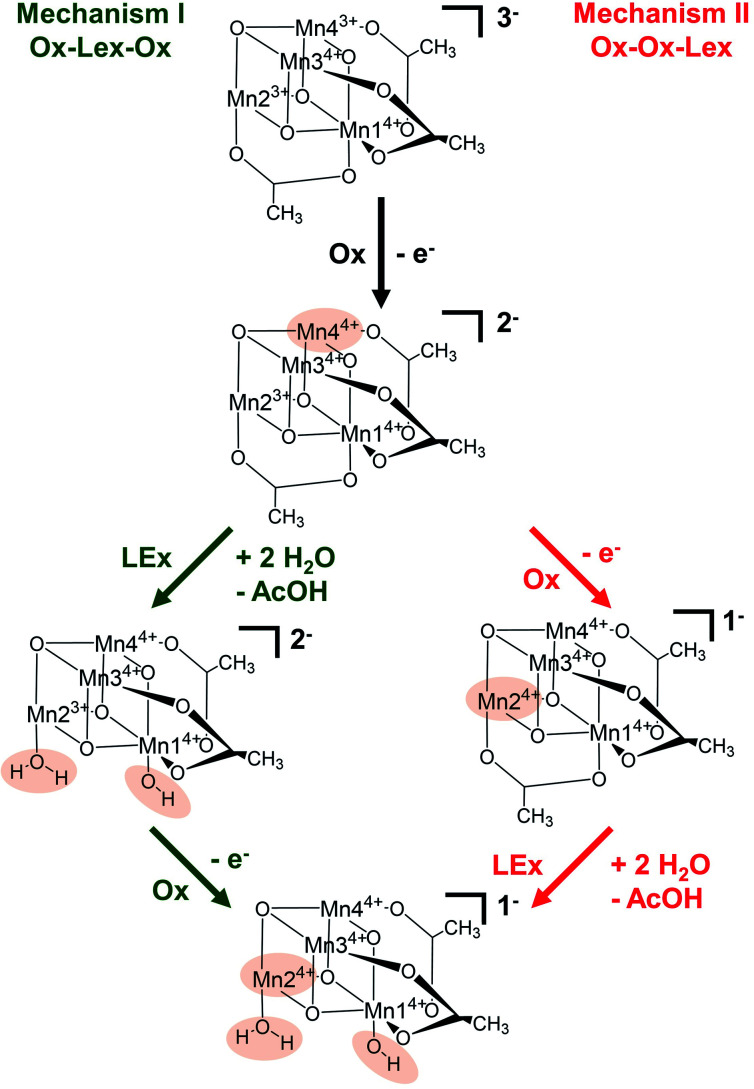
Schematic representation of the two proposed reaction mechanisms (I and II) involving oxidation (Ox) and ligand exchange (LEx) of the catalyst. Chemical changes relative to the previous step are highlighted in beige. Note that all calculations showed that the acetate ligand dissociates as a protonated acetic acid molecule due to the higher basicity of acetate compared with water. Thus, in both reactions the calculations show that the acetate is replaced by a H_2_O and an OH ligand.

Based on these experimental data, we now use theory to calculate reaction energies and activation barriers to unravel which of the two plausible mechanisms—proposed in Scheme 1— follows the first [Mn_2_^3+^Mn_2_^4+^] → [Mn^3+^Mn_3_^4+^] oxidation (Ox) step. In mechanism I (green in Scheme 1), the first oxidation is followed by the acetate ligand exchange (LEx) with water, after which the second [Mn^3+^Mn_2_^4+^] → [Mn_4_^4+^] oxidation step (Ox) takes place (globally summarized as Ox–LEx–Ox). In mechanism II (red), the first and second oxidations occur before the acetate ligand exchange (summarized as Ox–Ox–LEx). As the highest reactivity (based on turnover frequencies and electrochemical current densities) was observed in a 9 : 1 MeCN : H_2_O (v/v) mixture,^19^ these calculations were carried out considering the same solvent composition.

**Fig. 3:**
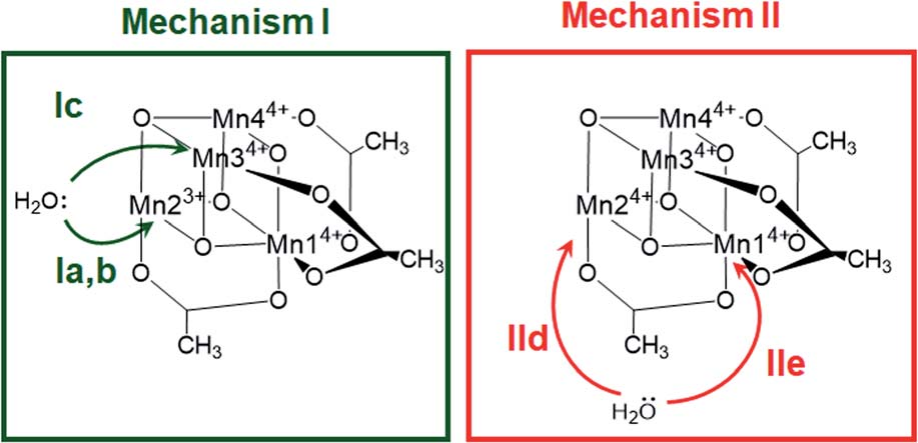
Possible water attack sites for the proposed ligand exchange pathways in mechanism I (Ox–LEx–Ox) and mechanism II (Ox–Ox–LEx), labelled (a)–(e).

**Fig. 4:**
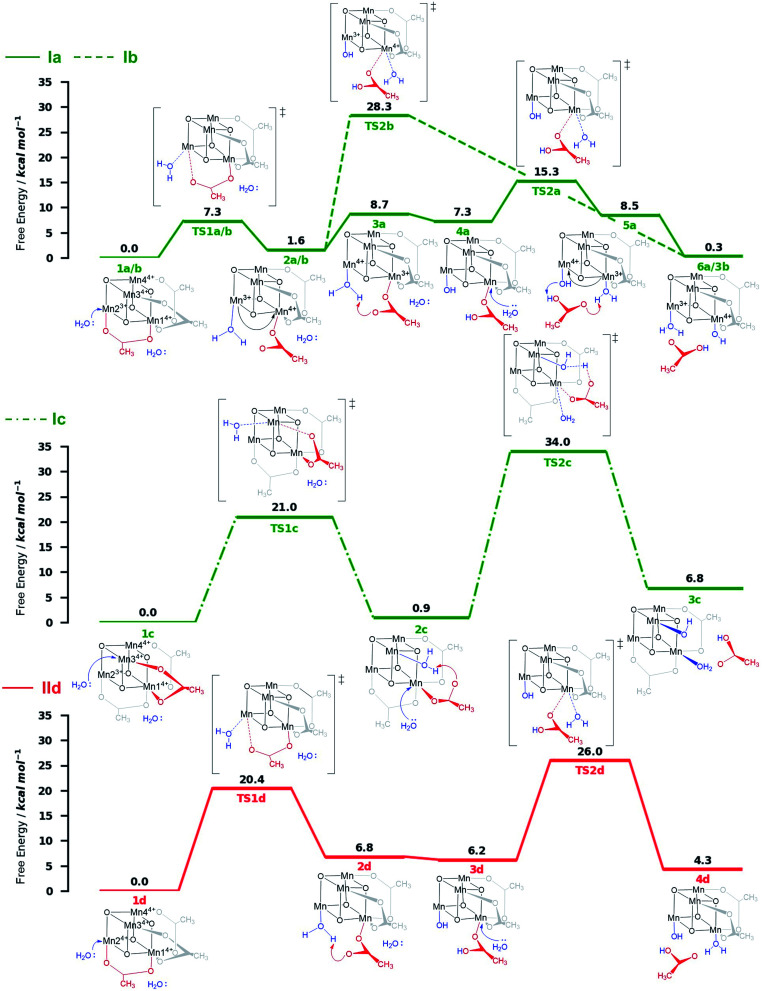
Energy diagrams (Δ*G* free energy in kcal mol^−1^) of the ligand exchange pathways studied at the B3LYP-D3/Def2-TZVP(Mn,V,O)-Def2-SVP(C,H)//B3LYP-D3/Def2-SVP level of theory with implicit solvent in a 9 : 1 MeCN : H_2_O ratio. Oxidation numbers at the Mn-cubane are only shown where they change. Pathways Ia–Ic (green) correspond to ligand exchange reactions which occur before the second oxidation step (Ox–LEx–Ox); pathway IId (red) corresponds to ligand exchange which occurs after the second oxidation process (Ox–Ox–LEx). The structures below and above each energy diagram are reaction intermediates and transition states, respectively. Energies are given relative to the reactant species.

The X-ray structrure of the native catalyst^19^ has an idealized *C*_3v_ symmetry (due to the appearence of delocalized electrons on the Mn-centres), so that in the [Mn_4_O_4_] cubane (Fig. 1b), Mn1 is located on the *C*_3_ axis, while the three manganese centers Mn2, Mn3 and Mn4 are symmetry equivalent. However, when considering the electronic structure of the one-electron-oxidized species [Mn^3+^Mn_3_^4+^], the crystallographic *C*_3v_ symmetry^19^ is lowered due to the presence of three Mn^4+^ centers and one Mn^3+^ center (which could correspond to any of the four Mn positions). Furthermore, Mn^3+^ (d^4^) ions in an octahedral environment show Jahn–Teller (JT) distortions, with one of the axes – the JT axis – elongated and the other two shortened. Such JT distortions have previously been identified in the analogous S_2_ oxidation state of the OEC,^66,67^ and in other related model complexes.^28,61,68^ The JT distortion implies that for each possible position of the Mn^3+^ ion, there are three possible JT axes, giving rise to three different structures. Due to the symmetry equivalence of Mn2, Mn3 and Mn4, we only have to consider localization of the Mn^3+^ ion at the positions Mn1 or Mn2, resulting in six possible structures (Fig. S6†). Accordingly, we performed geometry optimizations based on the six possible locations of the JT axes. In all cases, the geometries converged towards a minimum where the Mn^3+^ was localized at the Mn2 position, and the JT axis pointed along the Mn–acetate bond (red axis in Fig. 1b). This minimum energy structure was used hereafter. However, identifying the preferred JT axis localization does not yield any information on the acetate-to-water ligand exchange step, which could still occur at any of the Mn centers. Accordingly, in both mechanisms I and II, several ligand exchange pathways need to be considered (depicted in Fig. 3 and labelled (a)–(e)). The resulting intermediates and transition states (TS), together with their associated Gibbs free energies (Table S2†) and spin assignments (Tables S3 and S4†), are shown in Fig. 4.

Both ligand exchange pathways Ia and Ib involve the nucleophilic attack of the first water molecule at the Mn2 center, and the second water molecule at the Mn1 center. The first step of pathway Ia (Fig. 4, green solid line) is the ligand exchange of one oxygen donor of the acetate group by a water molecule at Mn2^3+^ (step 1a → 2a) with an activation barrier of 7.3 kcal mol^−1^ (TS1a/b). A sequential intramolecular electron transfer (ET) from Mn2 to Mn1 takes place (2a → 3a), followed by a proton transfer (PT) from the coordinated water molecule to the acetate ligand (3a → 4a), resulting in the formation of a Mn–OH group. This PT reaction is not water mediated (unlike analogous PT reactions reported by Maksimchuk *et al.*^69^) as the second water molecule does not actively participate in it. The second acetate ligand exchange at Mn1^3+^ (4a → 5a) represents the rate determining step of this path, with an activation Gibbs free energy of 8.0 kcal mol^−1^ (TS2a). Finally, an intramolecular concerted electron-proton transfer (5a → 6a) gives rise to a more stable product featuring one Mn–OH_2_ and one Mn–OH group, where the JT axis is again localized on the Mn2 atom.

The initial step of pathway Ib (Fig. 4, green dashed line) is identical to pathway Ia (1b → 2b). However, the second ligand exchange occurs at Mn1^4+^ (2b → 3b) *via* TS2b to give 3b, which is identical to 6a. Although thermodynamically possible, this pathway is kinetically unfavorable with an activation barrier of 26.7 kcal mol^−1^ and is therefore disregarded.

Pathway Ic (Fig. 4, green dashed-dotted line) starts with the nucleophilic attack of a water molecule at a Mn^4+^ atom (either the symmetry equivalent Mn3/Mn4 or Mn1). The attack on Mn3/4 gives an activation barrier of 21 kcal mol^−1^ (1c → TS1c → 2c). During the second ligand exchange, a proton is transferred from a Mn-coordinated water molecule to the departing acetate ligand, forming a Mn^4+^–OH moiety. This step (2c → 3c) presents a prohibitively high activation barrier of 33.1 kcal mol^−1^ (TS2c). An additional ligand exchange pathway initiated by water attacking the Mn1 center has not been explored further as no transition states for this pathway were found.

Pathways IId and IIe describe the ligand exchange within mechanism II, *i.e.*, after the oxidation to [Mn_4_^4+^] has taken place. The first ligand exchange can either occur at Mn2^4+^ (pathway IId, Mn3 and Mn4 are symmetry equivalent to Mn2) or at Mn1^4+^ (pathway IIe). The first ligand exchange in pathway IId (1d → 2d) has a barrier of 20.3 kcal mol^−1^ (TS1d) and is thus the rate limiting step. This step is followed by PT from the coordinated water to the acetate ligand (2d → 3d), generating again an OH group coordinated to Mn2. The second ligand exchange occurs at Mn1 with an activation barrier of 19.8 kcal mol^−1^ (TS2d). The structures and energetics of pathway IIe are very similar to those of pathway IId and thus are only shown in the ESI (Fig. S7†).

Pathways Ia and Ib are the most thermodynamically favorable ones with a total reaction free energy of 0.3 kcal mol^−1^. However, pathway Ib presents a very large energy barrier of 26.7 kcal mol^−1^ in the process 2b→3b *via* TS2b, in contrast to the 8 kcal mol^−1^ of pathway Ia in the step 4a → 5a *via* TS2a. The low barriers found only in pathway Ia appear to result from both acetate ligand exchange steps taking place at the Mn^3+^ centers, mirroring behavior observed in the OEC.^28^ Furthermore, all substitutions at Mn^3+^ centers occur along their JT axis, so that it is plausible to infer that the lower energy barriers evidenced for these ligand substitutions are associated with the elongated Mn^3+^–O bond being easier to cleave, with respect to its shorter Mn^4+^–O counterpart (see also Table S5†).

The calculations thus suggest that ligand exchange in mechanism I (Ox–LEx–Ox) is most likely to occur *via* pathway Ia. Concerning mechanism II (Ox–Ox–LEx), pathways IId and IIe have reaction free energies of 4.3 and 3.9 kcal mol^−1^, respectively, and are less favorable than pathways Ia and Ib, albeit still thermodynamically feasible. However, their large activation barriers (>20 kcal mol^−1^) make them both kinetically unfavorable. Interestingly, all pathways investigated end in a species with a water and an OH group as ligands.

**Fig. 5:**
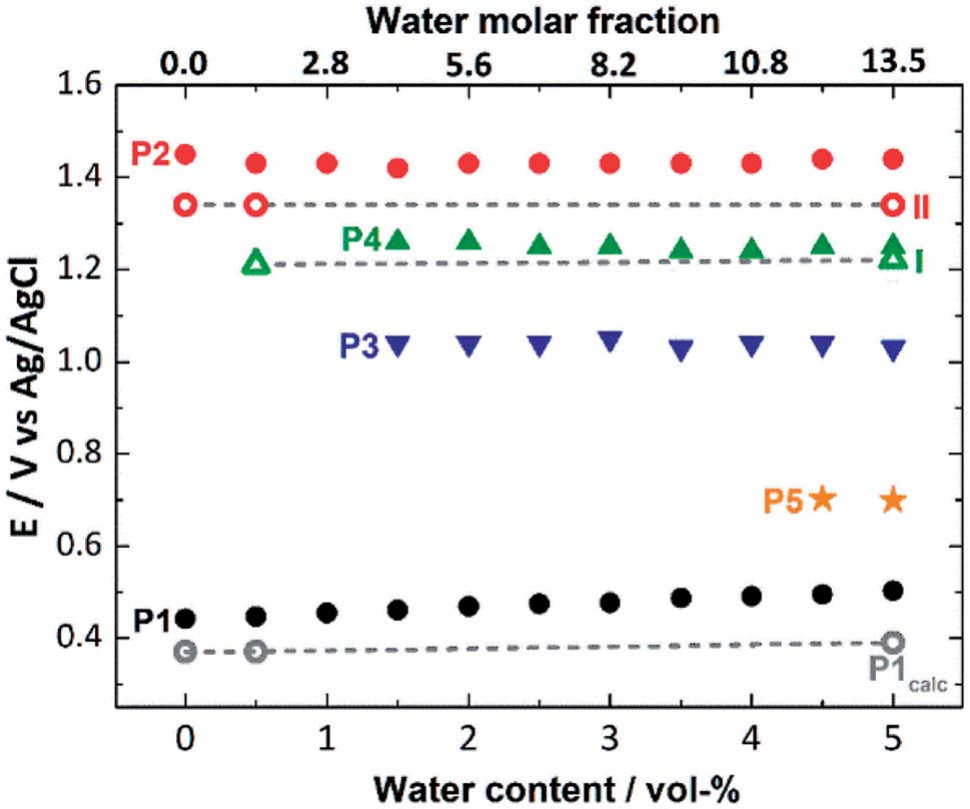
Comparison of experimental (full symbols) and theoretical (empty symbols, linked by gray dashed lines) oxidation potentials for the processes P1 to P5 (also see Fig. 2), depending on the water content (0–5 vol%) of the SWV electrolyte.

Next, we used this detailed theoretical assessment to gain further insight into the catalyst activation by correlating theoretically calculated redox potentials with the experimental SWV data measured at different solvent water contents (Fig. 5, Table S6†). Since P1 can be unambiguously assigned to the [Mn_2_^3+^Mn_2_^4+^] → [Mn^3+^Mn_3_^4+^] oxidation, the value of the redox potential obtained theoretically provides an estimate of the errors involved. Based on three calculated MeCN : H_2_O ratios, the mean error in P1 is 0.09 V, which shows good agreement between theory and experiment and is in line with the literature.^70–72^ In addition, the shift of the experimental redox-potential to more positive values (80 mV when going from 0 vol% to 5 vol% water content, Fig. 5, black dots) is qualitatively reproduced by theory, where a shift of 20 mV (between 0 vol% and 5 vol% water content) is calculated (Fig. 5, gray dashed line).

The experimental P2 signal (1.45 V at 0 vol% water content) can then be assigned to the [Mn^3+^Mn_3_^4+^] → [Mn_4_^4+^] transition, as acetate-to-water ligand exchange cannot occur under water-free conditions. Comparison with the theoretical potential calculated in mechanism II (Ox–Ox–LEx, 1.34 V) gives a good agreement (mean error: 0.1 V). In the presence of water, the Ox–LEx–Ox mechanism I becomes possible, and our redox calculations predict that the [Mn^3+^Mn_3_^4+^] → [Mn_3_^4+^] oxidation following the most favorable pathway Ia in mechanism I (Scheme 1) will occur at a potential of 1.22 V. This is in line with the appearance of the new oxidation peak P4 (1.25 V, mean error 0.03 V) in the presence of water (Fig. 5). To gain further insights into the correlation between peaks P2 and P4, variable-frequency SWV analyses were used. This method (in analogy to scan rate variation in CV) allows frequency-dependent study of the kinetics of electron transfer processes, while retaining the advantages of SWV over CV. Variation of the SWV frequency between 5 and 50 Hz showed that with increasing frequency (analogous to higher scan rate in CV), process P2 is partially recovered (ESI, Fig. S4†). This is in line with the interpretation that at higher SWV frequencies, the oxidation step to [Mn_4_^4+^] (ESI, Fig. S3,† step 2a) can now compete with the acetate-to-water ligand exchange (ESI, Fig. S3,† step 2b). Note that this suggestion is also in agreement with our initial hypothesis that under the given electrochemical conditions, ligand exchange occurs only after the [Mn_2_^3+^Mn_2_^4+^] → [Mn^3+^Mn_3_^4+^] oxidation. This is also supported by IR-spectroscopic data described earlier (Fig. 2a) and will in the future be studied by *in situ* spectro-electrochemistry. Finally, the experimentally observed redox peaks P3 and P5 are at this point assigned to intermediate, electrode-surface-adsorbed species (ESI, Fig. S5†), which will be analyzed in more detail in a follow-up study.

In summary, these data support the hypothesis that mechanism I is most favorable at the given experimental conditions following these steps: (i) [Mn_2_^3+^Mn_2_^4+^] → [Mn^3+^Mn_3_^4+^] oxidation, (ii) acetate for water ligand exchange and deprotonation of one water ligand, according to pathway Ia, and (iii) [Mn^3+^Mn_3_^4+^] → [Mn_4_^4+^] oxidation, *i.e.* Ox–LEx–Ox.

## Conclusions

This work presents theoretical and experimental insights into the redox and ligand exchange processes of the initial activation steps of the molecular manganese vanadium oxide water oxidation catalyst [Mn_4_V_4_O_17_(AcO_3_)]^3−^. Extensive DFT calculations were used to model redox properties and the ligand exchange pathways of the catalyst prior to entering the water oxidation cycle. We demonstrate that the currently most plausible activation mechanism involves a one-electron oxidation of the catalyst [Mn_2_^3+^Mn_2_^4+^] → [Mn^3+^Mn_3_^4+^], followed by acetate-to-water ligand exchange and a second one-electron oxidation from [Mn^3+^Mn_3_^4+^] to [Mn_4_^4+^]. The ligand exchange pathways investigated show that the kinetically most favorable pathway is characterized by two nucleophilic attacks of water molecules along the Jahn–Teller axis of Mn^3+^ centers. Theory predicts that the oxidized species [Mn_4_V_4_O_17_(OAc)_2_(H_2_O)(OH)]^−^ features one H_2_O and one OH ligand, as a result of deprotonation of one water ligand and proton transfer to the leaving acetate group during the ligand exchange. The final products derived from the proposed mechanism allowed us to calculate redox potentials that are in excellent agreement with electrochemical measurements carried out at various solvent compositions, supporting our speciation assignment. This study therefore offers a comprehensive understanding of the complex interplay between electronic structure, redox chemistry, and acid–base ligand exchange in molecular metal-oxide water oxidation catalysts. Further, we provide a blueprint for how experimental electrochemistry and IR spectroscopy together with theoretical calculations can be utilized to explore complex mechanisms in multi-electron catalytic systems. We expect similar studies to guide the design of advanced mixed metal oxide water oxidation catalysts. Future work will focus on studying the speciation of the catalyst at higher oxidation states as a starting point for the water oxidation cycle.

## Data availability

Inputs/outputs as well as experimental results are available upon reasonable request.

## Author contributions

LG and CS designed the study. GC, DHC, and LS performed and analyzed the calculations. JJN, PM and LG supervised the theoretical work. IT synthesised the compound. JK, RS, SK and BM provided IR-spectroscopic analyses. IT, GL, CK and CS provided electrochemical analyses. GC, DHC and IT wrote the original draft. CS and LG reviewed and edited the manuscript. All authors discussed and commented on the manuscript. LG, CS, BM and CK acquired funding.

## Conflicts of interest

There are no conflicts to declare.

## Supplementary Material

SC-012-D1SC03239A-s001

SC-012-D1SC03239A-s002
