# Supplementary material for: Activation by oxidation and ligand exchange in a molecular manganese vanadium oxide water oxidation catalyst
Source: Chem Sci. 2021 Aug 30;12(39):12918–27. doi: 10.1039/d1sc03239a (PMC8513927; doi:10.1039/d1sc03239a)
Supplement: SC-012-D1SC03239A-s001 [file SC-012-D1SC03239A-s001.pdf]

Electronic Supplementary Material (ESI) for Chemical Science.
This journal is © The Royal Society of Chemistry 2021

Cartesian coordinates of the optimized structures in the reaction pathways calculations

Pathways 1a and 1b

Structure 1a (1b)

Mn	-2.33297	-0.75125	0.05851
Mn	-0.4819	1.07876	1.24049
Mn	0.21922	-1.80297	0.5246
Mn	-0.23434	0.0292	-1.63689
O	-1.00398	-1.58131	-0.93729
O	-1.12928	-0.75406	1.4512
O	0.87857	-0.18189	-0.14435
O	-1.51227	0.83302	-0.42197
H	-4.32601	0.97586	-3.71277
H	-3.43211	-0.12728	-4.80086
C	-2.92547	-0.23901	-2.71136
C	-3.91965	-0.04474	-3.82084
O	-1.71506	0.0507	-2.95548
O	-3.38477	-0.64151	-1.59347
H	-4.74878	-0.75881	-3.72732
H	-4.95064	2.67427	1.1499
C	-4.77351	2.03313	2.0304
C	-3.52143	1.27263	1.70373
O	-2.40647	1.74653	2.03276
O	-3.68505	0.1789	1.04885
H	-5.63148	1.35929	2.15563
H	-4.63024	2.66576	2.91634
O	-0.87766	-3.37826	1.0424
O	-2.92823	-2.52369	0.60477
O	0.7105	0.89501	2.66479
V	1.94315	-0.30577	2.62791

O	2.4753	-0.54883	4.09203
O	1.28556	-1.79433	2.01311
O	0.41773	1.69072	-2.06995
V	1.19314	2.68374	-0.87806
O	0.25441	2.68549	0.57894
O	1.28116	4.15674	-1.42967
O	2.84012	-2.79279	-2.78111
V	2.17209	-1.7814	-1.77929
O	1.40977	-2.72913	-0.5302
O	0.95536	-0.9052	-2.67485
O	3.50013	-0.72177	-1.16564
O	5.24585	0.98609	-0.08967
V	3.71515	0.62672	-0.06589
O	2.8532	2.06775	-0.57758
O	3.32142	0.21524	1.58738
H	-2.07069	-5.56053	1.41516
H	-3.10566	-4.58154	2.49941
C	-2.78481	-4.72805	1.45383
C	-2.14071	-3.45171	0.99047
H	-3.67742	-4.94492	0.85095
H	-2.22073	3.70209	1.6956
O	-1.87866	4.37138	1.0758
H	-1.01523	3.96807	0.84004
O	-3.29106	2.91141	-0.8836
H	-2.67477	2.15408	-0.84401
H	-2.84661	3.55151	-0.2886

Structure TS1a (TS1b)

Mn	-2.386610	0.462897	0.146418
Mn	-0.360935	-1.549454	0.762416
Mn	-0.188343	0.352017	-1.620641
Mn	0.085719	1.459619	1.012536
O	-1.078578	1.613716	-0.491307
O	-1.454434	-0.895980	-0.733952
O	0.880912	0.171847	-0.091876
O	-1.210519	0.071990	1.514157
H	-4.094729	3.651935	2.768795
H	-2.508786	4.253029	3.403505
C	-2.358121	2.795438	1.830472
C	-3.064990	3.933783	2.512143
O	-1.097879	2.739207	1.947091
O	-3.079450	1.987738	1.160884
H	-3.099988	4.778048	1.802242
H	-5.470725	-2.832869	0.239141
C	-5.304272	-2.371132	1.227265
C	-3.933952	-1.742553	1.188263
O	-2.938611	-2.333285	1.603994
O	-3.900037	-0.561844	0.644152
H	-5.346870	-3.154245	1.995931
H	-6.086475	-1.616607	1.389256
O	-1.613891	0.636288	-2.955437
O	-3.356826	0.858368	-1.535024
O	0.569919	-2.731074	-0.352551
V	1.504171	-2.231001	-1.692541
O	1.737467	-3.459864	-2.655503
O	0.612710	-1.003111	-2.544955
O	1.139830	1.101490	2.466378

V	1.941557	-0.432407	2.610748
O	0.809663	-1.668629	2.238172
O	2.441271	-0.612310	4.096268
O	2.606715	3.661241	-1.747238
V	2.048737	2.330238	-1.121386
O	0.922364	1.653928	-2.273417
O	1.204263	2.759820	0.337480
O	3.472797	1.270320	-0.812384
O	5.376261	-0.520788	-0.250601
V	3.815886	-0.330637	-0.179762
O	3.361902	-0.496863	1.502936
O	3.108011	-1.589476	-1.163070
H	-4.643980	1.547522	-3.613660
H	-3.268675	1.264846	-4.761035
C	-3.786525	0.907629	-3.861653
C	-2.842794	0.809494	-2.697736
H	-4.155112	-0.116480	-4.042610
H	-1.728200	-3.522897	2.155284
O	-0.825035	-3.852420	1.993330
H	-0.273391	-3.398909	2.652981
O	-3.627677	-2.105622	-1.982007
H	-2.756463	-1.845486	-1.618733
H	-4.195723	-1.420760	-1.598397

Structure 2a (2b)

Mn	-2.301614	0.140928	0.070257
Mn	-0.145100	-0.713100	-1.679092
Mn	0.036978	1.707844	0.173831
Mn	-0.042854	-0.918998	1.370527

O	-1.089775	0.693688	1.360395
O	-1.145688	0.905083	-1.173147
O	0.988906	0.085723	0.161683
O	-1.219671	-1.350434	-0.140974
H	-3.983026	-3.150979	2.848754
H	-3.077654	-2.536872	4.265954
C	-2.701908	-1.524764	2.402267
C	-3.616311	-2.242340	3.356181
O	-1.457428	-1.718295	2.522329
O	-3.261641	-0.795693	1.518306
H	-4.483902	-1.612985	3.597893
H	-6.142012	-0.716277	-0.762319
C	-5.564940	-1.644213	-0.637874
C	-4.236295	-1.502503	-1.328453
O	-3.736651	-2.406603	-1.995084
O	-3.652757	-0.332126	-1.183616
H	-6.115863	-2.519080	-1.006261
H	-5.362316	-1.755918	0.440341
O	-1.314280	3.174671	0.266323
O	-3.171884	1.889984	0.326109
O	0.990635	0.258466	-2.830456
V	1.988874	1.528526	-2.240948
O	2.448578	2.439008	-3.456466
O	1.068011	2.503671	-1.119732
O	0.909576	-2.470496	1.141543
V	1.863163	-2.722946	-0.302615
O	0.935840	-2.272197	-1.675761
O	2.239132	-4.261131	-0.408592
O	2.478969	1.689416	3.777096

V	1.999922	1.075949	2.399534
O	1.057245	2.311462	1.591436
O	0.983896	-0.297918	2.778375
O	3.503598	0.620423	1.498431
O	5.539691	-0.155802	-0.067287
V	3.957708	-0.101653	-0.043610
O	3.394657	-1.756661	-0.203431
O	3.465702	0.876189	-1.416008
H	-2.894301	5.129479	0.041242
H	-4.308292	4.054517	-0.329159
C	-3.449655	4.230127	0.335372
C	-2.568368	3.012386	0.305038
H	-3.833590	4.356613	1.361394
H	-2.335327	-1.937939	-2.698194
O	-1.465961	-1.655065	-3.117058
H	-1.018583	-2.490544	-3.337975
O	-5.679655	1.742377	-1.103654
H	-4.915863	1.176635	-1.321780
H	-5.484362	1.935279	-0.173537

Structure 3a

Mn	-2.427323	0.077708	0.151554
Mn	-0.160664	-0.770865	-1.428272
Mn	-0.007799	1.687542	0.098821
Mn	0.006331	-0.808930	1.461740
O	-0.878724	0.807535	1.467423
O	-1.213746	0.800663	-1.132024
O	0.917862	-0.005695	-0.044254
O	-1.204918	-1.381326	0.057086

H	-3.524427	-3.056491	3.491943
H	-2.735420	-2.042524	4.730680
C	-2.535817	-1.359135	2.698594
C	-3.319485	-2.017131	3.801122
O	-1.267732	-1.490855	2.754588
O	-3.184507	-0.759296	1.793012
H	-4.277936	-1.502655	3.950561
H	-6.446339	-0.960490	-1.198208
C	-5.863512	-1.885082	-1.308906
C	-4.402873	-1.559630	-1.507193
O	-3.646616	-2.402059	-2.043882
O	-4.002200	-0.421827	-1.084736
H	-6.246162	-2.506832	-2.130373
H	-5.954754	-2.461637	-0.371999
O	-1.305372	3.137745	0.199570
O	-3.187140	1.898295	0.405408
O	0.831974	0.025731	-2.760567
V	1.851097	1.377205	-2.381527
O	2.210195	2.150761	-3.709901
O	0.963491	2.413376	-1.341366
O	0.983218	-2.410863	1.289646
V	1.865889	-2.725061	-0.146006
O	0.834851	-2.323309	-1.485228
O	2.231948	-4.258972	-0.211695
O	2.721866	1.957042	3.535636
V	2.199381	1.242264	2.229908
O	1.221040	2.347414	1.326404
O	1.235707	-0.124277	2.673881
O	3.626791	0.715080	1.259314

O	5.549998	-0.176866	-0.377770
V	3.982658	-0.108006	-0.242786
O	3.388205	-1.754320	-0.219169
O	3.382061	0.798877	-1.611322
H	-2.859996	5.092738	-0.150030
H	-4.325054	4.030164	-0.299101
C	-3.410380	4.242972	0.272990
C	-2.568997	2.996919	0.291800
H	-3.701816	4.480301	1.309988
H	-2.362286	-1.905242	-2.353783
O	-1.418139	-1.543091	-2.714694
H	-0.972046	-2.309426	-3.119131
O	-5.820722	1.737462	-0.887772
H	-5.159816	1.058216	-1.128800
H	-5.523911	1.948984	0.010484

Structure 4a

Mn	-2.405485	0.207517	-0.028762
Mn	-0.137511	-0.904372	-1.428577
Mn	0.051221	1.704876	-0.114631
Mn	-0.100773	-0.628244	1.482213
O	-0.936299	1.009005	1.287643
O	-1.126607	0.748265	-1.318570
O	0.916078	0.001541	-0.016053
O	-1.261039	-1.302641	0.083529
H	-4.560449	-1.828152	3.367138
H	-3.106911	-2.124069	4.404590
C	-2.742481	-0.973034	2.615716
C	-3.623696	-1.405107	3.756255

O	-1.489870	-1.143090	2.762385
O	-3.314154	-0.450052	1.611120
H	-3.874866	-0.507736	4.347043
H	-6.197522	-1.376495	-0.519609
C	-5.619998	-2.240127	-0.870942
C	-4.263740	-1.788184	-1.314795
O	-3.448524	-2.706587	-1.714404
O	-3.947175	-0.579735	-1.283566
H	-6.138151	-2.743511	-1.702318
H	-5.503535	-2.975884	-0.059182
O	-1.221281	3.207213	-0.221133
O	-3.152209	2.043473	-0.038916
O	0.986997	-0.256465	-2.750909
V	2.020489	1.093370	-2.440952
O	2.482492	1.720674	-3.815619
O	1.114724	2.258237	-1.559961
O	0.821025	-2.267316	1.531097
V	1.770686	-2.749868	0.185542
O	0.838445	-2.465408	-1.245229
O	2.091904	-4.292586	0.297619
O	2.584666	2.246219	3.433589
V	2.105596	1.420322	2.176811
O	1.220853	2.464616	1.118168
O	1.073081	0.136162	2.705431
O	3.568828	0.761181	1.349072
O	5.548364	-0.346247	-0.074676
V	3.978102	-0.218874	-0.040934
O	3.329705	-1.836297	0.109428
O	3.489284	0.559398	-1.527040

H	-2.736226	5.153152	-0.841032
H	-4.263766	4.185367	-0.699437
C	-3.277997	4.394046	-0.261621
C	-2.486503	3.118661	-0.175055
H	-3.424295	4.774424	0.763779
H	-2.536710	-2.290199	-2.040064
O	-1.321105	-1.707653	-2.611794
H	-0.823999	-2.405879	-3.073654
O	-5.906901	1.429493	-0.640677
H	-5.364783	0.785913	-1.129063
H	-5.220934	1.836044	-0.086619

Structure TS2a

Mn	-2.427846	0.754219	-0.235528
Mn	-0.262749	-0.652917	-1.524147
Mn	0.234971	1.839718	-0.068703
Mn	-0.404606	-0.491458	1.397700
O	-0.959695	1.264085	1.219713
O	-0.981143	1.137669	-1.389577
O	0.816015	0.017518	0.013252
O	-1.534436	-0.909608	-0.106957
H	-4.890395	-1.515826	2.862963
H	-3.759066	-1.221021	4.227516
C	-3.158282	-0.417133	2.314406
C	-4.208746	-0.784703	3.327214
O	-1.971928	-0.818365	2.539186
O	-3.545266	0.252497	1.308647
H	-4.799774	0.107497	3.582362
H	-5.010408	-3.046777	0.760245

C	-4.045801	-3.369026	0.348752
C	-3.673568	-2.538143	-0.846245
O	-2.756876	-3.090272	-1.606975
O	-4.163225	-1.430984	-1.055144
H	-4.060437	-4.440378	0.102070
H	-3.257908	-3.209811	1.104656
O	-0.761218	3.522374	-0.169436
O	-2.868790	2.689194	-0.197367
O	1.013870	-0.145610	-2.756067
V	2.242460	0.988269	-2.291954
O	2.913152	1.585813	-3.591326
O	1.487382	2.253934	-1.413066
O	0.218408	-2.261948	1.442684
V	1.185826	-2.847746	0.148214
O	0.456267	-2.352187	-1.333448
O	1.236256	-4.426855	0.202204
O	2.551248	1.835272	3.663969
V	2.037899	1.146046	2.339639
O	1.415626	2.359730	1.278499
O	0.773419	0.031819	2.738705
O	3.435334	0.289954	1.578477
O	5.305018	-1.082155	0.245219
V	3.775019	-0.709679	0.183813
O	2.878139	-2.208206	0.245735
O	3.517716	0.164595	-1.310374
H	-1.856936	5.746529	-0.604151
H	-3.541081	5.086831	-0.681826
C	-2.573995	5.041605	-0.163202
C	-2.029434	3.638554	-0.189549

H	-2.732546	5.320670	0.892656
H	-2.309103	-2.379178	-2.180688
O	-1.488182	-1.179783	-2.794196
H	-1.012874	-1.339001	-3.628196
O	-4.076338	0.866729	-2.377283
H	-4.068135	-0.104410	-2.230408
H	-4.890366	1.138744	-1.922479

Structure 5a

Mn	-2.400414	0.989523	-0.389360
Mn	-0.265663	-0.551676	-1.574946
Mn	0.316794	1.892151	-0.068804
Mn	-0.540319	-0.406733	1.334124
O	-0.967945	1.382658	1.158413
O	-0.873990	1.283030	-1.463418
O	0.778359	0.042200	0.015328
O	-1.618545	-0.737340	-0.228022
H	-5.085322	-1.391684	2.386455
H	-4.097068	-1.208111	3.876826
C	-3.327422	-0.249867	2.096338
C	-4.462543	-0.699151	2.976486
O	-2.165571	-0.684522	2.389780
O	-3.618833	0.506374	1.125633
H	-5.087966	0.165391	3.244044
H	-4.173827	-3.748284	1.005575
C	-3.231471	-3.745588	0.443195
C	-3.339557	-2.901557	-0.793342
O	-2.416492	-3.173553	-1.688346
O	-4.190582	-2.025043	-0.921135

H	-2.909707	-4.769141	0.203570
H	-2.437258	-3.279152	1.053413
O	-0.569778	3.639067	-0.201383
O	-2.718653	2.945556	-0.391977
O	1.084006	-0.103728	-2.753424
V	2.362320	0.936823	-2.211563
O	3.133975	1.502277	-3.468989
O	1.655844	2.242355	-1.350538
O	-0.037278	-2.222449	1.381927
V	0.971303	-2.849107	0.138598
O	0.381261	-2.278257	-1.374189
O	0.927236	-4.429897	0.165950
O	2.444854	1.705792	3.769146
V	1.955744	1.064137	2.411585
O	1.460767	2.326486	1.340120
O	0.612501	0.019562	2.734301
O	3.338921	0.142107	1.703062
O	5.177770	-1.334942	0.440160
V	3.675662	-0.872695	0.318370
O	2.691586	-2.315076	0.347598
O	3.531001	0.015791	-1.183056
H	-1.466708	5.942555	-0.633619
H	-3.157703	5.389482	-0.967331
C	-2.277942	5.273191	-0.319514
C	-1.820701	3.839012	-0.314190
H	-2.573427	5.536177	0.710673
H	-2.188091	-2.369464	-2.258897
O	-1.484024	-1.036548	-2.870612
H	-0.998298	-1.178487	-3.702124

O	-4.060728	0.471885	-1.700831
H	-4.092514	-0.522895	-1.598052
H	-4.895199	0.764847	-1.294471

Structure 6a

Mn	-2.205175	1.056922	-0.318742
Mn	-0.295042	-0.653616	-1.647501
Mn	0.485000	1.852780	-0.128212
Mn	-0.488218	-0.372674	1.381404
O	-0.958689	1.444556	1.033977
O	-0.768741	1.233429	-1.487900
O	0.894939	0.057192	0.192292
O	-1.611989	-0.686003	-0.176904
H	-5.043830	-1.047136	2.516439
H	-4.111785	-0.692644	4.009612
C	-3.259398	0.012939	2.155411
C	-4.435929	-0.291164	3.041174
O	-2.154482	-0.543403	2.438904
O	-3.475864	0.780230	1.167979
H	-5.054771	0.607328	3.174373
H	-5.002915	-3.651263	0.808559
C	-4.041684	-3.642745	0.275066
C	-3.989068	-2.539389	-0.743918
O	-3.431171	-2.667875	-1.827317
O	-4.570859	-1.428736	-0.342847
H	-3.846065	-4.613612	-0.197644
H	-3.245678	-3.438188	1.011552
O	-0.362276	3.641501	-0.376665
O	-2.527032	2.984989	-0.477562

O	1.169846	-0.275041	-2.770981
V	2.455376	0.725904	-2.244067
O	3.262203	1.242991	-3.501147
O	1.803877	2.089783	-1.389678
O	-0.056437	-2.157732	1.473905
V	0.856521	-2.911085	0.201183
O	0.227118	-2.435650	-1.316529
O	0.740774	-4.481179	0.344562
O	2.539059	1.705373	3.689532
V	1.990854	1.047774	2.366755
O	1.551821	2.350380	1.297687
O	0.578684	0.125570	2.804484
O	3.332281	0.019390	1.725855
O	5.112204	-1.570319	0.526634
V	3.639010	-1.037146	0.359365
O	2.596798	-2.441186	0.348922
O	3.585427	-0.186982	-1.167153
H	-1.228079	5.934285	-0.947673
H	-2.928699	5.381715	-1.229672
C	-2.045426	5.299865	-0.581258
C	-1.603346	3.865162	-0.480017
H	-2.330479	5.637302	0.429867
H	-2.511514	-1.566186	-2.712890
O	-1.711424	-1.227903	-3.199350
H	-1.352100	-2.033017	-3.611297
O	-3.506864	0.605750	-1.552755
H	-4.272362	-0.638572	-0.906919
H	-3.970892	1.388421	-1.894766

Structure TS2b

Mn	2.376486	0.538929	0.211162
Mn	0.233919	-0.854887	1.606897
Mn	-0.162618	1.793305	0.152875
Mn	0.341058	-0.552097	-1.435687
O	1.175403	1.138053	-1.059756
O	1.028963	0.932418	1.424175
O	-0.891700	0.100745	-0.184090
O	1.443996	-1.066219	0.068487
H	3.743553	-0.232362	-4.411079
H	5.078574	-0.437647	-3.220206
C	3.080977	-0.503766	-2.405103
C	4.072397	-0.767905	-3.506005
O	1.899267	-0.934430	-2.592647
O	3.490855	0.120547	-1.381788
H	4.074555	-1.843732	-3.737471
H	4.800590	-2.444491	-0.988780
C	4.007493	-2.982050	-0.452659
C	3.705704	-2.343230	0.886398
O	3.233701	-3.059161	1.796538
O	3.912446	-1.086724	1.008523
H	4.264684	-4.044141	-0.333787
H	3.080559	-2.918702	-1.046276
O	0.958954	3.408999	0.359382
O	2.992822	2.443668	0.145514
O	-1.094210	-0.240119	2.792025
V	-2.209056	0.972572	2.327979
O	-2.860557	1.603075	3.620481
O	-1.361932	2.222915	1.457377

O	-0.391496	-2.218171	-1.539389
V	-1.382541	-2.823357	-0.237256
O	-0.618793	-2.498613	1.257525
O	-1.550259	-4.384105	-0.406450
O	-2.347166	2.090502	-3.582268
V	-1.874305	1.318298	-2.294299
O	-1.157320	2.497947	-1.231302
O	-0.663120	0.173766	-2.802680
O	-3.351066	0.536015	-1.607423
O	-5.337546	-0.744766	-0.364483
V	-3.788571	-0.487069	-0.251292
O	-3.013881	-2.054359	-0.315025
O	-3.520872	0.292887	1.291627
H	3.859335	4.796339	-0.098685
H	2.226630	5.575141	-0.023335
C	2.878523	4.796384	0.394917
C	2.225697	3.444903	0.280891
H	3.026255	5.006723	1.468062
H	2.317664	-2.179065	2.666861
O	1.609730	-1.574679	3.095024
H	1.162341	-2.123854	3.759474
O	3.592834	1.015389	2.222130
H	3.871970	0.066600	2.179734
H	4.367871	1.507078	1.903594

Pathway 1c

Structure 1c

Mn	-2.251896	-0.746986	-0.032492

Mn	-0.358977	0.776744	-1.552053
Mn	-0.244810	0.466415	1.516043
Mn	0.323717	-1.831990	-0.105534
O	-0.973285	-1.282844	1.198785
O	-1.462725	0.922025	0.085814
O	0.939277	-0.087678	0.177125
O	-0.986863	-1.066094	-1.325505
H	-1.898180	-5.693754	-0.684268
H	-2.898271	-5.242885	0.732348
C	-2.000562	-3.589470	-0.243163
C	-2.615589	-4.959879	-0.295853
O	-0.737918	-3.508195	-0.266006
O	-2.811517	-2.606362	-0.152499
H	-3.529584	-4.941397	-0.905893
H	-5.208006	1.785256	-2.082211
C	-4.626029	1.267606	-2.862187
C	-3.371415	0.766510	-2.203780
O	-2.253556	1.206017	-2.542542
O	-3.567114	-0.106216	-1.269410
H	-4.392214	1.949576	-3.688983
H	-5.226941	0.416727	-3.217583
O	-1.811179	0.798459	2.709388
O	-3.395851	-0.248081	1.479126
O	0.323331	2.494494	-1.269978
V	1.181521	2.877302	0.171869
O	1.237216	4.443882	0.340549
O	0.365583	2.191376	1.549125
O	1.460932	-2.171488	-1.498515
V	2.141737	-0.870842	-2.433647

O	0.908142	0.260880	-2.825416
O	2.747593	-1.460349	-3.765065
O	2.788114	-1.930059	3.455471
V	2.159480	-1.206746	2.208518
O	0.892984	-0.163203	2.809302
O	1.466078	-2.448677	1.198197
O	3.504047	-0.300800	1.416217
O	5.288500	1.123746	0.032529
V	3.763937	0.736532	0.023843
O	3.463426	-0.085430	-1.491116
O	2.864339	2.236806	0.111602
H	-3.663207	1.428848	4.284759
H	-4.726259	1.589981	2.828165
C	-4.082714	0.911749	3.413430
C	-3.004703	0.448237	2.478285
H	-4.696503	0.053240	3.725193
H	-2.517691	3.374131	-1.122764
O	-2.726723	3.259409	-0.181924
H	-2.193190	2.462786	0.047159
H	-4.343018	2.561144	0.174555
H	-4.827977	1.130482	0.341676
O	-5.153740	2.043273	0.384116

Structure TS1c

Mn	2.383940	-0.422095	0.172671
Mn	0.179025	0.523360	1.775435
Mn	0.305437	0.905974	-1.278029
Mn	-0.032656	-1.767081	-0.243522
O	1.259435	-0.795898	-1.255051

O	1.401035	1.162342	0.286197
O	-0.847071	-0.059972	-0.224432
O	1.029068	-1.136760	1.233239
H	2.528562	-5.424994	-0.060194
H	3.449456	-4.685730	-1.401489
C	2.423335	-3.281146	-0.194562
C	3.168821	-4.578090	-0.339726
O	1.160896	-3.320110	-0.326378
O	3.115835	-2.237009	0.016893
H	4.087732	-4.561727	0.261391
H	3.735993	1.269952	4.716309
C	4.141774	1.068526	3.716565
C	3.051341	0.632064	2.775465
O	1.859430	0.896064	3.042936
O	3.454456	0.035349	1.708324
H	4.943929	0.318520	3.759550
H	4.568964	1.999832	3.307094
O	2.551742	0.944144	-2.725785
O	3.748589	0.297235	-0.928374
O	-0.745601	2.149904	1.822284
V	-1.621478	2.712955	0.460232
O	-1.885504	4.259078	0.621138
O	-0.657922	2.453540	-0.969047
O	-1.236051	-2.535222	0.910726
V	-2.147807	-1.591230	2.044374
O	-1.120631	-0.412194	2.752381
O	-2.724677	-2.535254	3.169905
O	-2.221054	-1.326442	-3.976749
V	-1.782849	-0.841973	-2.546991

O	-0.622926	0.457720	-2.777643
O	-0.999295	-2.182709	-1.769972
O	-3.283944	-0.308563	-1.699320
O	-5.408996	0.443831	-0.249169
V	-3.846630	0.290958	-0.147522
O	-3.521258	-0.820345	1.157507
O	-3.197005	1.868370	0.250815
H	4.789411	1.211550	-3.908560
H	5.461354	1.789775	-2.346312
C	4.943778	0.957848	-2.851745
C	3.618031	0.722078	-2.164954
H	5.582006	0.065899	-2.758826
H	0.791229	3.532875	-1.388583
O	1.609659	3.352486	-1.880522
H	2.305468	3.323117	-1.192184
H	2.542432	2.420806	0.493625
H	3.998111	2.327604	0.127836
O	3.333138	2.994737	0.366104

Structure 2c

Mn	2.291227	0.162244	0.031717
Mn	0.015395	1.887579	0.137645
Mn	0.029265	-0.662318	-1.523777
Mn	0.062635	-0.948815	1.318346
O	1.231856	-1.361558	-0.183962
O	1.129642	0.906376	-1.204214
O	-0.974851	-0.195537	-0.041708
O	1.067696	0.695280	1.288638
H	3.109917	-2.385566	4.290403

H	3.859987	-3.198849	2.888194
C	2.706143	-1.500786	2.370499
C	3.615302	-2.217661	3.330144
O	1.458678	-1.680208	2.506743
O	3.260086	-0.795584	1.469336
H	4.549516	-1.657102	3.467761
H	4.777254	3.725288	-0.275820
C	3.921288	4.045401	0.335389
C	2.853049	2.984510	0.282038
O	1.645911	3.297424	0.251414
O	3.301925	1.776448	0.282690
H	3.527191	5.013313	-0.000494
H	4.265831	4.140291	1.379064
O	3.357949	-2.407450	-2.148200
O	3.633151	-0.397418	-1.226094
O	-1.113926	2.594533	-1.179246
V	-2.037258	1.563248	-2.193148
O	-2.510732	2.370516	-3.464110
O	-1.042192	0.233137	-2.707456
O	-0.985793	-0.293959	2.666408
V	-1.989312	1.108779	2.422904
O	-1.116663	2.316528	1.573381
O	-2.421044	1.675387	3.831223
O	-2.245548	-4.193856	-0.411664
V	-1.849305	-2.677333	-0.265282
O	-0.902504	-2.257767	-1.664455
O	-0.855520	-2.540592	1.156606
O	-3.396929	-1.751026	-0.157378
O	-5.534512	-0.152317	0.008815

V	-3.961214	-0.098085	0.004174
O	-3.474796	0.624022	1.519830
O	-3.490575	0.917707	-1.340371
H	5.740861	-2.922446	-1.306390
H	6.030669	-1.176455	-0.878042
C	5.327874	-2.020799	-0.836074
C	4.019837	-1.622108	-1.460420
H	5.125742	-2.218984	0.230415
H	2.075034	-1.754271	-2.649396
O	1.214548	-1.310631	-3.005105
H	0.720860	-2.024030	-3.449390
H	5.699911	1.528644	-0.010163
H	5.071840	0.912214	-1.210215
O	5.898443	1.357713	-0.943941

Structure TS2c

Mn	2.359732	0.449310	-0.038245
Mn	-0.097468	1.927829	-0.273914
Mn	0.146909	-0.892285	-1.382588
Mn	0.220177	-0.596448	1.476985
O	1.439197	-1.176444	0.053008
O	1.133650	0.792202	-1.397408
O	-0.867648	-0.197287	0.026691
O	1.115257	1.109661	1.079394
H	3.362287	-1.145150	4.638077
H	4.114674	-2.210574	3.419770
C	2.868356	-0.715609	2.589532
C	3.836079	-1.167356	3.647448
O	1.640505	-0.967902	2.778377

O	3.354637	-0.143640	1.566807
H	4.744471	-0.551273	3.628540
H	4.111836	4.334945	-1.528885
C	3.460505	4.532207	-0.662736
C	2.536648	3.360716	-0.464088
O	1.301678	3.520154	-0.473686
O	3.126951	2.226934	-0.287862
H	2.890373	5.456153	-0.820661
H	4.109365	4.633360	0.221674
O	2.983469	-3.050244	-1.867258
O	3.699645	-0.949843	-1.791227
O	-1.293590	2.260538	-1.662868
V	-2.155428	0.972193	-2.402723
O	-2.712296	1.450373	-3.799328
O	-1.058506	-0.347222	-2.654156
O	-0.849777	0.251485	2.687594
V	-1.980703	1.483920	2.196136
O	-1.230433	2.550407	1.080287
O	-2.424554	2.299781	3.470545
O	-1.838977	-4.276286	0.512954
V	-1.568302	-2.736729	0.333465
O	-0.675787	-2.542188	-1.137811
O	-0.560025	-2.233396	1.670258
O	-3.183423	-1.933114	0.298066
O	-5.456353	-0.521702	0.223050
V	-3.895188	-0.338157	0.151005
O	-3.439486	0.708131	1.476296
O	-3.536957	0.420709	-1.382723
H	4.886081	-3.666503	-0.327343

H	5.861302	-2.252905	-0.877787
C	4.883050	-2.581480	-0.494120
C	3.787886	-2.169043	-1.451659
H	4.716717	-2.050593	0.456644
H	1.988310	-2.366053	-2.436682
O	1.171599	-1.727372	-2.825017
H	0.535486	-2.317816	-3.266014
H	4.872567	1.511567	-0.524129
H	4.807606	0.073065	-1.082567
O	5.138891	0.603364	-0.323251

Structure 3c

Mn	2.212408	1.041452	-0.247527
Mn	-0.430684	2.061569	-0.113733
Mn	0.230427	-0.583752	-1.515365
Mn	0.481631	-0.466464	1.347574
O	1.612938	-0.730835	-0.184501
O	0.828271	1.285273	-1.434755
O	-0.802956	-0.199063	0.035073
O	1.000948	1.383860	1.063876
H	4.186533	-1.104540	3.841167
H	5.037782	-1.402499	2.287775
C	3.303063	-0.217944	2.083305
C	4.494607	-0.654341	2.889305
O	2.165213	-0.657405	2.418323
O	3.544370	0.550790	1.094905
H	5.170585	0.196438	3.056544
H	3.326861	5.252089	-1.336429
C	2.731338	5.221691	-0.410296

C	2.014495	3.898498	-0.325464
O	0.770346	3.862159	-0.254439
O	2.787424	2.864706	-0.335845
H	2.016306	6.053806	-0.394303
H	3.432623	5.308621	0.434639
O	2.660577	-3.074093	-1.664066
O	4.334745	-1.796069	-0.860893
O	-1.781034	2.254558	-1.401114
V	-2.405291	0.886117	-2.226978
O	-3.169933	1.368905	-3.521399
O	-1.093545	-0.153476	-2.707680
O	-0.612713	0.050574	2.727431
V	-1.986128	1.073833	2.412104
O	-1.544204	2.361195	1.365931
O	-2.489854	1.676811	3.781653
O	-0.895868	-4.416087	0.203561
V	-0.941618	-2.841589	0.150133
O	-0.275396	-2.360222	-1.374732
O	0.055265	-2.253823	1.453968
O	-2.684073	-2.380645	0.317071
O	-5.157449	-1.393688	0.459035
V	-3.660077	-0.926626	0.317561
O	-3.325463	0.097856	1.695355
O	-3.555182	-0.033111	-1.185570
H	3.622028	-4.884093	-0.211560
H	4.880817	-3.854011	0.584423
C	3.857130	-3.888800	0.190575
C	3.647889	-2.816325	-0.839729
H	3.150049	-3.680577	1.012352

H	2.326784	-2.267991	-2.175084
O	1.576940	-0.963646	-2.820350
H	1.194664	-1.019951	-3.712427
H	2.796837	0.099318	-2.393919
H	3.899313	-0.352228	-1.407671
O	3.446229	0.489091	-1.733371

Pathway 2d

Structure 1d (1e)

Mn	0.214227	1.544347	-0.218081
Mn	2.429107	-0.150152	0.145406
Mn	0.108054	-0.758994	1.608084
Mn	0.241329	-1.178253	-1.304816
O	1.347328	0.404824	-1.266039
O	1.233040	0.763405	1.225756
O	-0.781138	-0.117476	-0.017368
O	1.258527	-1.577029	0.289606
H	4.453168	-3.315374	-2.417214
H	3.355741	-3.085629	-3.829679
C	2.926436	-1.880841	-2.101309
C	3.884698	-2.582160	-3.012053
O	1.683134	-2.021240	-2.314874
O	3.440970	-1.185341	-1.157641
H	4.599925	-1.843737	-3.406753
H	4.463510	-1.126892	3.990265
C	3.545930	-1.725503	3.909762
C	2.697823	-1.216239	2.786186
O	1.434786	-1.276634	2.931635

O	3.304914	-0.784256	1.749590
H	2.983623	-1.724334	4.851800
H	3.830647	-2.762725	3.662218
H	4.117767	4.121041	0.640059
O	1.631566	2.891150	-0.340844
C	3.810378	3.835817	-0.380684
C	2.881779	2.669289	-0.262139
H	4.709455	3.535781	-0.937720
H	3.312220	4.687556	-0.860070
O	3.412471	1.524269	-0.064804
O	-0.952259	0.241717	2.710035
V	-1.901409	1.555480	2.058375
O	-2.359759	2.499199	3.233002
O	-0.855171	2.451083	0.969303
O	-0.796052	-2.659840	-1.071382
V	-1.853230	-2.779100	0.318351
O	-0.928034	-2.256628	1.708566
O	-2.282621	-4.281920	0.516294
O	-2.024968	1.458175	-3.993711
V	-1.688793	0.889070	-2.563874
O	-0.725131	2.061287	-1.688655
O	-0.703905	-0.547009	-2.732401
O	-3.217818	0.527725	-1.691685
O	-5.393390	-0.080571	-0.229788
V	-3.820524	-0.093803	-0.157083
O	-3.320112	-1.762163	0.102734
O	-3.346834	0.940033	1.191647
O	5.962590	0.258453	-0.683108
H	5.347597	0.965055	-0.432163

H	5.359613	-0.381395	-1.092621
O	-0.348375	5.109203	0.120631
H	-0.696300	4.285202	0.505745
H	0.543797	4.828392	-0.131939

Structure TS1d

Mn	0.207394	-1.285281	1.011636
Mn	2.371316	0.464766	0.172677
Mn	0.277487	-0.004432	-1.630757
Mn	-0.108637	1.619207	0.796391
O	1.143218	0.332490	1.569596
O	1.461869	-1.020598	-0.484192
O	-0.801105	-0.049787	0.013143
O	1.060161	1.459989	-0.697029
H	3.905715	4.450342	1.493963
H	2.284206	5.160875	1.887253
C	2.266843	3.074243	1.391855
C	2.934441	4.280871	1.976634
O	0.990666	3.039407	1.451507
O	3.004113	2.161473	0.912208
H	3.102707	4.076671	3.048211
H	3.396475	0.112622	-4.751233
C	3.892946	0.300652	-3.792171
C	2.911226	0.331757	-2.664336
O	1.686549	0.100177	-2.929530
O	3.377798	0.563126	-1.503716
H	4.440854	1.255264	-3.815945
H	4.614949	-0.497721	-3.554845
H	4.954952	-3.144325	2.409018

O	2.678467	-2.160106	1.750612
C	5.081466	-2.227002	1.820646
C	3.750079	-1.606413	1.508554
H	5.562553	-2.450294	0.854498
H	5.717688	-1.505429	2.354791
O	3.824509	-0.437707	0.928025
O	-0.514227	-1.499410	-2.317513
V	-1.482869	-2.511896	-1.296029
O	-1.669661	-3.912298	-1.976467
O	-0.562913	-2.705607	0.173654
O	-1.241492	2.661975	-0.178855
V	-2.038248	2.076940	-1.608861
O	-0.873624	1.130426	-2.480344
O	-2.504167	3.289266	-2.489640
O	-2.639757	0.121776	4.038747
V	-2.111863	0.087694	2.562037
O	-0.941401	-1.190819	2.400200
O	-1.236884	1.548275	2.227782
O	-3.478789	-0.165802	1.432349
O	-5.417265	-0.583170	-0.381144
V	-3.868885	-0.373499	-0.265477
O	-3.454215	1.067216	-1.168853
O	-3.081987	-1.786172	-0.950418
H	1.573301	-3.423676	2.295019
O	0.613430	-3.484269	2.462402
H	0.261675	-3.979459	1.703823
H	6.243201	-1.441184	-1.543078
O	5.312554	-1.713287	-1.492366
H	4.886842	-0.949819	-1.066891

Structure 2d

Mn	0.073067	-0.578717	-1.592387
Mn	2.334343	0.118568	0.074154
Mn	0.010932	1.703314	0.243973
Mn	0.077002	-1.050503	1.292791
O	1.243062	-1.365402	-0.252480
O	1.174046	0.964270	-1.130642
O	-0.902580	-0.005084	-0.038007
O	1.088288	0.578794	1.368565
H	4.488298	-1.891233	3.441894
H	3.037128	-2.717902	4.148928
C	2.688184	-1.684232	2.300376
C	3.573619	-2.459462	3.227006
O	1.433076	-1.875113	2.399771
O	3.253553	-0.917808	1.456885
H	3.854984	-3.391545	2.706859
H	4.410174	3.976741	0.173168
C	3.452195	4.162266	0.678186
C	2.582573	2.953749	0.516319
O	1.321352	3.119911	0.478758
O	3.178497	1.832350	0.438455
H	2.952483	5.058240	0.288135
H	3.642536	4.300155	1.756353
H	5.938101	-2.598861	-1.699832
O	3.453025	-2.172734	-2.298170
C	5.493314	-1.807934	-1.082972
C	4.127116	-1.436966	-1.571573
H	6.126394	-0.910184	-1.040036

H	5.375118	-2.172853	-0.047549
O	3.690997	-0.265270	-1.171643
O	-1.023585	2.582392	-0.983287
V	-1.983125	1.675875	-2.124197
O	-2.408671	2.621785	-3.311433
O	-0.945032	0.407973	-2.740179
O	-0.991491	-0.482716	2.661695
V	-2.005190	0.929770	2.472075
O	-1.050881	2.150658	1.665739
O	-2.430457	1.457291	3.895728
O	-2.252788	-4.206331	-0.696731
V	-1.888264	-2.693385	-0.442277
O	-0.910814	-2.106548	-1.765340
O	-0.892340	-2.570214	0.985792
O	-3.399991	-1.730163	-0.285264
O	-5.548582	-0.120236	-0.070145
V	-3.973395	-0.081965	-0.050026
O	-3.481434	0.539615	1.520978
O	-3.452316	0.984498	-1.353111
H	2.171098	-1.565261	-2.718680
O	1.297400	-1.088201	-3.035491
H	1.571694	-0.302137	-3.546290
O	5.854185	1.668728	-0.755827
H	5.666355	1.702066	0.195222
H	5.057258	1.215347	-1.080657

Structure 3d

Mn	0.036119	-1.001175	-1.454572
Mn	2.308118	0.217597	-0.138824

Mn	-0.062798	1.711614	-0.272364
Mn	0.167929	-0.639741	1.473045
O	1.287547	-1.332932	0.015494
O	1.080683	0.661917	-1.460124
O	-0.906087	-0.013495	-0.007620
O	1.134092	0.976653	1.048075
H	4.124578	-0.265051	4.073572
H	3.353765	-1.864977	4.338792
C	2.855006	-0.872349	2.499050
C	3.820500	-1.210368	3.592736
O	1.622936	-1.091822	2.698759
O	3.346330	-0.362157	1.435343
H	4.718309	-1.676552	3.162210
H	3.468453	4.464976	-1.685079
C	3.283522	4.325832	-0.605916
C	2.476298	3.073949	-0.445063
O	1.210395	3.180011	-0.474317
O	3.121227	1.981533	-0.319874
H	2.726559	5.194236	-0.230747
H	4.250387	4.218453	-0.096567
H	6.099508	-1.829872	-2.240098
O	3.385488	-2.591773	-1.931013
C	5.583445	-1.973004	-1.274342
C	4.140950	-1.665163	-1.483721
H	5.999580	-1.269009	-0.542452
H	5.712335	-3.021256	-0.971770
O	3.723855	-0.492030	-1.251586
O	-1.177055	2.197968	-1.631137
V	-2.155440	0.985046	-2.424244

O	-2.682787	1.560954	-3.794885
O	-1.105350	-0.370544	-2.741533
O	-0.838372	0.255617	2.708646
V	-1.920834	1.530148	2.194521
O	-1.061051	2.514355	1.034560
O	-2.295780	2.417662	3.442904
O	-2.121037	-4.276341	0.567019
V	-1.803598	-2.743033	0.371133
O	-0.926781	-2.527031	-1.113077
O	-0.741390	-2.205927	1.654355
O	-3.346665	-1.814225	0.353961
O	-5.541320	-0.262090	0.245524
V	-3.969012	-0.179666	0.166659
O	-3.430484	0.859820	1.481666
O	-3.555296	0.500566	-1.404942
H	2.410617	-2.246361	-2.203836
O	1.166738	-1.807353	-2.675671
H	1.233698	-1.255510	-3.477188
O	5.862523	0.809884	0.616813
H	5.141322	0.382786	1.109391
H	5.393823	1.106255	-0.179095

Structure TS2d

Mn	0.207394	-1.285281	1.011636
Mn	2.371316	0.464766	0.172677
Mn	0.277487	-0.004432	-1.630757
Mn	-0.108637	1.619207	0.796391
O	1.143218	0.332490	1.569596
O	1.461869	-1.020598	-0.484192

O	-0.801105	-0.049787	0.013143
O	1.060161	1.459989	-0.697029
H	3.905715	4.450342	1.493963
H	2.284206	5.160875	1.887253
C	2.266843	3.074243	1.391855
C	2.934441	4.280871	1.976634
O	0.990666	3.039407	1.451507
O	3.004113	2.161473	0.912208
H	3.102707	4.076671	3.048211
H	3.396475	0.112622	-4.751233
C	3.892946	0.300652	-3.792171
C	2.911226	0.331757	-2.664336
O	1.686549	0.100177	-2.929530
O	3.377798	0.563126	-1.503716
H	4.440854	1.255264	-3.815945
H	4.614949	-0.497721	-3.554845
H	4.954952	-3.144325	2.409018
O	2.678467	-2.160106	1.750612
C	5.081466	-2.227002	1.820646
C	3.750079	-1.606413	1.508554
H	5.562553	-2.450294	0.854498
H	5.717688	-1.505429	2.354791
O	3.824509	-0.437707	0.928025
O	-0.514227	-1.499410	-2.317513
V	-1.482869	-2.511896	-1.296029
O	-1.669661	-3.912298	-1.976467
O	-0.562913	-2.705607	0.173654
O	-1.241492	2.661975	-0.178855
V	-2.038248	2.076940	-1.608861

O	-0.873624	1.130426	-2.480344
O	-2.504167	3.289266	-2.489640
O	-2.639757	0.121776	4.038747
V	-2.111863	0.087694	2.562037
O	-0.941401	-1.190819	2.400200
O	-1.236884	1.548275	2.227782
O	-3.478789	-0.165802	1.432349
O	-5.417265	-0.583170	-0.381144
V	-3.868885	-0.373499	-0.265477
O	-3.454215	1.067216	-1.168853
O	-3.081987	-1.786172	-0.950418
H	1.573301	-3.423676	2.295019
O	0.613430	-3.484269	2.462402
H	0.261675	-3.979459	1.703823
H	6.243201	-1.441184	-1.543078
O	5.312554	-1.713287	-1.492366
H	4.886842	-0.949819	-1.066891

Structure 4d

Mn	-0.230285	0.406782	-1.658926
Mn	-2.262175	-1.117678	-0.292604
Mn	0.392919	-1.930736	0.050063
Mn	-0.627181	0.539026	1.269326
O	-1.653853	0.639666	-0.365462
O	-0.799859	-1.451249	-1.398395
O	0.755187	-0.035965	0.028009
O	-1.113756	-1.329128	1.113384
H	-5.388029	0.082917	2.710465
H	-4.434249	1.465538	3.394665

C	-3.455378	0.355941	1.832651
C	-4.684766	0.898212	2.490197
O	-2.333047	0.848190	2.175594
O	-3.626377	-0.546507	0.948540
H	-5.150599	1.566056	1.745845
H	-1.354251	-6.026612	-0.261968
C	-2.179865	-5.337778	-0.044107
C	-1.713486	-3.915723	-0.104227
O	-0.471603	-3.693820	0.024195
O	-2.614952	-3.019461	-0.253265
H	-2.542583	-5.522965	0.981614
H	-3.019403	-5.488949	-0.736538
H	-2.746998	4.911088	0.013892
O	-2.420607	3.109880	-1.692361
C	-3.105882	3.945899	0.398410
C	-3.315757	2.953852	-0.712823
H	-4.026107	4.074143	0.982728
H	-2.316539	3.531288	1.048504
O	-4.164661	2.085198	-0.707413
O	1.751297	-2.321505	-1.103163
V	2.459422	-1.047613	-2.072030
O	3.286728	-1.696970	-3.247009
O	1.139657	-0.119931	-2.749021
O	0.403777	0.175770	2.735550
V	1.823671	-0.835773	2.556671
O	1.380335	-2.206724	1.568509
O	2.264332	-1.353812	3.979365
O	0.775224	4.443865	-0.204853
V	0.881862	2.872894	-0.100244

O	0.344996	2.147399	-1.582315
O	-0.166661	2.300961	1.180919
O	2.595911	2.415483	0.222589
O	5.092790	1.476887	0.539418
V	3.605116	0.983451	0.370927
O	3.193556	0.066695	1.817384
O	3.546614	-0.014699	-1.082567
H	-2.343303	2.290955	-2.235483
O	-1.543639	0.708976	-2.949196
H	-1.182986	0.699656	-3.855073
O	-3.378490	-0.862903	-1.943484
H	-3.437940	-1.693864	-2.455230
H	-2.813000	-0.234961	-2.502579

Pathway IIe

Structure TS1e

Mn	-0.346926	1.413863	-0.638637
Mn	-2.488612	-0.437103	-0.015454
Mn	-0.114666	-1.487781	-1.103579
Mn	-0.226495	-0.431665	1.661910
O	-1.456936	0.858758	0.859483
O	-1.376466	-0.020613	-1.453492
O	0.721385	-0.128713	0.000196
O	-1.236100	-1.630841	0.494609
H	-4.597787	-1.581377	3.763999
H	-3.164631	-1.407747	4.861508
C	-2.829637	-0.822619	2.824580
C	-3.718636	-0.974516	4.019326

O	-1.578303	-0.745319	3.026493
O	-3.394694	-0.751862	1.681972
H	-4.064180	0.035307	4.300966
H	-3.776369	-3.052470	-3.582209
C	-3.446164	-3.593912	-2.678802
C	-2.636862	-2.638053	-1.858193
O	-1.372816	-2.669255	-1.994792
O	-3.275673	-1.837292	-1.100073
H	-2.838331	-4.455631	-2.982183
H	-4.338389	-3.909527	-2.121360
H	-2.985714	4.614439	-2.370904
O	-1.450656	2.808775	-1.200095
C	-3.231031	4.330913	-1.335181
C	-2.743031	2.932974	-1.072029
H	-4.312962	4.402656	-1.167247
H	-2.672217	5.001972	-0.664415
O	-3.499791	2.012573	-0.760109
O	0.889767	-1.126589	-2.559722
V	1.820461	0.362737	-2.633063
O	2.241317	0.637735	-4.126991
O	0.752228	1.622059	-2.099663
O	0.860463	-1.812415	2.156300
V	1.912393	-2.564580	0.977782
O	0.968862	-2.811735	-0.477894
O	2.381987	-3.963805	1.528566
O	1.957405	3.222472	2.687637
V	1.641150	2.019090	1.721152
O	0.630903	2.582260	0.419477
O	0.690302	0.824670	2.585527

O	3.177632	1.322183	1.109031
O	5.377421	0.142541	0.094894
V	3.805367	0.050835	0.061561
O	3.355505	-1.546611	0.651876
O	3.296031	0.274437	-1.607397
O	-5.285436	0.125227	-0.221795
H	-5.002220	1.036817	-0.420541
H	-5.352949	0.114063	0.746579
H	-0.166896	4.281759	0.428795
O	-0.433393	5.212515	0.297716
H	-0.330794	5.303046	-0.662151

Structure 2e

Mn	0.228839	1.497666	0.392467
Mn	2.595513	-0.161083	0.232773
Mn	0.269225	-1.303631	1.310670
Mn	0.497670	-0.690814	-1.560162
O	1.518717	0.847702	-0.915245
O	1.327923	0.317417	1.511103
O	-0.617204	-0.217687	-0.037688
O	1.489716	-1.570582	-0.158121
H	5.134209	-1.746350	-3.077174
H	3.783182	-2.006027	-4.258782
C	3.222763	-1.019768	-2.438172
C	4.218321	-1.327103	-3.514213
O	1.990803	-1.079400	-2.746575
O	3.682148	-0.689573	-1.295641
H	4.471402	-0.373948	-4.010041
H	3.868391	-2.138550	4.212409

C	3.674194	-2.812061	3.359974
C	2.841025	-2.055278	2.371445
O	1.578770	-2.193423	2.443422
O	3.456971	-1.303193	1.546436
H	3.134691	-3.696114	3.722798
H	4.637647	-3.088518	2.911001
H	0.363434	5.207436	0.096586
O	1.292425	2.966041	0.815375
C	1.099983	4.861811	-0.641475
C	1.899602	3.739766	-0.058718
H	1.754145	5.668333	-0.997021
H	0.535724	4.445644	-1.493224
O	3.081035	3.535144	-0.355792
O	-0.886290	-0.816998	2.637831
V	-1.957136	0.543991	2.409168
O	-2.527248	0.999769	3.806260
O	-0.989250	1.834645	1.735877
O	-0.430213	-2.226894	-1.898720
V	-1.493658	-2.899530	-0.683816
O	-0.659507	-2.812146	0.848424
O	-1.782965	-4.410249	-1.029720
O	-1.932420	2.523424	-3.309091
V	-1.579713	1.518297	-2.145987
O	-0.743685	2.381560	-0.882939
O	-0.455414	0.318860	-2.740911
O	-3.088193	0.763627	-1.523591
O	-5.247990	-0.455106	-0.473016
V	-3.680612	-0.385452	-0.327012
O	-3.051371	-2.000554	-0.627142

O	-3.325299	0.137139	1.318006
O	3.858471	1.313491	0.586150
H	3.556159	2.242694	0.218039
H	4.743754	1.142107	0.211276
O	-0.820892	4.627697	2.183037
H	-1.027720	3.703790	1.943526
H	0.131899	4.558091	2.347843

Structure 3e

Mn	0.292419	1.363304	0.782636
Mn	2.574419	-0.262767	-0.003490
Mn	0.209484	-1.576666	0.828373
Mn	0.338064	-0.145022	-1.728544
O	1.481012	1.082423	-0.734346
O	1.375285	-0.136652	1.436187
O	-0.674441	-0.070696	-0.055707
O	1.310350	-1.447510	-0.727257
H	4.788014	-0.961705	-3.815665
H	3.355903	-0.799031	-4.916232
C	2.973398	-0.351212	-2.852815
C	3.880723	-0.387187	-4.044596
O	1.724909	-0.242911	-3.076994
O	3.517721	-0.396611	-1.703578
H	4.172087	0.653970	-4.266688
H	3.732233	-3.562282	3.114201
C	3.565248	-3.848789	2.061546
C	2.758750	-2.758893	1.423184
O	1.492441	-2.829912	1.552764
O	3.391105	-1.829220	0.830885

H	3.008385	-4.795000	2.044271
H	4.538155	-3.948500	1.563368
H	1.158994	5.277718	1.606436
O	1.496821	2.670908	1.521171
C	1.547139	4.896987	0.650305
C	2.135123	3.548125	0.857943
H	2.267571	5.593138	0.204952
H	0.679793	4.758550	-0.018682
O	3.280022	3.293956	0.357620
O	-0.847800	-1.459007	2.322967
V	-1.810860	-0.033200	2.595680
O	-2.279582	0.007259	4.100265
O	-0.775976	1.343323	2.271765
O	-0.736505	-1.447433	-2.438998
V	-1.777847	-2.402235	-1.413451
O	-0.857871	-2.819113	0.010724
O	-2.192992	-3.721172	-2.172554
O	-1.962241	3.566273	-2.295912
V	-1.620064	2.242833	-1.508375
O	-0.669135	2.646418	-0.095737
O	-0.608032	1.228628	-2.497322
O	-3.143479	1.415560	-1.030026
O	-5.322369	0.065599	-0.201141
V	-3.747880	0.003994	-0.165568
O	-3.260510	-1.481660	-0.969690
O	-3.255861	0.002038	1.526224
O	3.857045	0.904441	0.670246
H	3.552082	2.266256	0.501300
H	4.745531	0.696760	0.326112

O	1.327680	0.504248	4.170180
H	0.663286	1.177534	3.957781
H	1.478190	0.121729	3.286587

Structure 4e

Mn	0.442239	1.882421	-0.157488
Mn	-2.222555	1.082636	-0.278149
Mn	-0.275677	-0.614429	-1.568252
Mn	-0.529416	-0.411743	1.366151
O	-1.002717	1.426615	1.027003
O	-0.831974	1.262797	-1.482379
O	0.786195	0.005258	0.025581
O	-1.655222	-0.680475	-0.196709
H	-5.048873	-1.174987	2.448967
H	-4.133866	-0.815081	3.951175
C	-3.290742	-0.065928	2.119696
C	-4.463149	-0.409793	2.986641
O	-2.165425	-0.574948	2.418302
O	-3.515826	0.693942	1.122020
H	-5.102583	0.473555	3.122588
H	-5.180915	-3.556394	0.506463
C	-4.137535	-3.695060	0.196926
C	-3.761925	-2.696309	-0.855475
O	-2.727899	-3.049198	-1.575855
O	-4.369696	-1.631572	-0.985213
H	-3.958371	-4.723578	-0.145713
H	-3.478433	-3.506724	1.062631
O	-0.394093	3.643715	-0.338169
O	-2.556827	2.982259	-0.407667

O	1.085385	-0.248517	-2.728299
V	2.416784	0.751159	-2.232685
O	3.188124	1.245739	-3.507446
O	1.743682	2.126552	-1.404036
O	-0.088828	-2.168787	1.462413
V	0.896430	-2.887197	0.217044
O	0.273847	-2.347198	-1.297590
O	0.783250	-4.452299	0.298944
O	2.467439	1.737777	3.713162
V	1.972423	1.067952	2.383039
O	1.502009	2.315849	1.266374
O	0.564788	0.098726	2.730669
O	3.303494	0.073678	1.710075
O	5.129687	-1.488845	0.525752
V	3.650411	-0.993914	0.362146
O	2.618790	-2.407644	0.398609
O	3.546154	-0.151198	-1.173838
H	-1.283778	5.930639	-0.908895
H	-3.003741	5.384957	-1.083210
C	-2.081378	5.301975	-0.492918
C	-1.639601	3.872092	-0.417247
H	-2.296342	5.636665	0.536583
H	-2.349534	-2.288133	-2.119803
O	-1.493556	-1.087595	-2.834669
H	-1.024045	-1.381914	-3.637396
O	-3.622499	0.697935	-1.618924
H	-3.906587	-0.282656	-1.493845
H	-4.427080	1.235182	-1.486222

Cartesian coordinates of the optimized structures in the redox potential calculations

[$Mn^{3+}{}_2Mn^{4+}{}_2$] eps=36.6

Mn	-0.29262	-1.28095	1.25451
O	-1.38299	-1.40117	-0.37340
Mn	-2.48102	0.02547	0.01432
O	-1.33764	0.37438	1.41249
Mn	-0.19548	1.59569	0.43424
O	-1.34322	1.03233	-1.02281
Mn	-0.29979	-0.47570	-1.72453
O	0.86471	0.20958	0.05640
C	-4.35705	-0.91005	-3.68927
H	-5.01182	-0.02586	-3.74065
H	-3.95227	-1.13697	-4.68423
H	-4.97288	-1.75330	-3.33679
C	-3.24280	-0.65907	-2.69465
O	-3.64280	-0.33532	-1.51708
O	-2.05592	-0.78197	-3.05795
C	-3.83008	3.95721	0.98873
H	-4.70373	3.63576	1.57420
H	-3.30362	4.77591	1.49665
H	-4.19561	4.31840	0.01249
C	-2.90433	2.78752	0.74375
O	-3.47367	1.68204	0.46087
O	-1.66422	2.99390	0.80977
C	-4.34173	-2.60892	2.79028
H	-3.93423	-3.34092	3.49978
H	-4.93479	-1.85377	3.33074
H	-5.01917	-3.11027	2.08077

C	-3.23163	-1.91934	2.02484
O	-2.04395	-2.21587	2.26412
O	-3.63582	-1.05539	1.16362
O	2.18500	-4.12255	-1.11952
V	1.69765	-2.66189	-0.72396
O	0.76735	-2.75145	0.71640
O	0.76180	-2.01500	-2.01001
O	3.16084	-1.62729	-0.44740
V	3.67511	-0.01262	-0.01038
O	5.25598	-0.06144	-0.02553
O	3.24768	1.19180	-1.20689
V	1.70449	1.86974	-1.89324
O	0.78552	0.68917	-2.74494
O	0.76153	2.71632	-0.70285
O	2.16309	2.95392	-2.95535
O	2.18203	1.06250	4.03618
V	1.71489	0.66070	2.57515
O	0.76393	1.98860	1.97961
O	0.79995	-0.79036	2.71920
O	3.25302	0.42675	1.63100

[$Mn^{3+}_2Mn^{4+}_2$] eps=36.82

Mn	-0.29263	-1.28087	1.25458
O	-1.38299	-1.40119	-0.37333
Mn	-2.48103	0.02547	0.01432
O	-1.33764	0.37446	1.41246
Mn	-0.19548	1.59571	0.43414
O	-1.34322	1.03228	-1.02288
Mn	-0.29980	-0.47580	-1.72450

O	0.86471	0.20958	0.05639
C	-4.35705	-0.91017	-3.68924
H	-5.01175	-0.02593	-3.74068
H	-3.95227	-1.13719	-4.68417
H	-4.97295	-1.75335	-3.33671
C	-3.24280	-0.65920	-2.69461
O	-3.64280	-0.33539	-1.51706
O	-2.05592	-0.78216	-3.05789
C	-3.83007	3.95724	0.98863
H	-4.70367	3.63580	1.57417
H	-3.30359	4.77597	1.49648
H	-4.19566	4.31839	0.01239
C	-2.90432	2.78756	0.74363
O	-3.47366	1.68207	0.46079
O	-1.66420	2.99395	0.80959
C	-4.34173	-2.60885	2.79035
H	-3.93422	-3.34069	3.50000
H	-4.93496	-1.85369	3.33061
H	-5.01901	-3.11040	2.08082
C	-3.23162	-1.91925	2.02493
O	-2.04393	-2.21574	2.26424
O	-3.63580	-1.05533	1.16367
O	2.18499	-4.12262	-1.11927
V	1.69765	-2.66193	-0.72378
O	0.76735	-2.75142	0.71657
O	0.76181	-2.01512	-2.00988
O	3.16084	-1.62732	-0.44727
V	3.67511	-0.01262	-0.01039
O	5.25598	-0.06144	-0.02553

O	3.24768	1.19171	-1.20698
V	1.70449	1.86962	-1.89335
O	0.78551	0.68901	-2.74499
O	0.76154	2.71628	-0.70302
O	2.16309	2.95374	-2.95553
O	2.18202	1.06274	4.03611
V	1.71488	0.66086	2.57510
O	0.76392	1.98873	1.97949
O	0.79995	-0.79019	2.71923
O	3.25302	0.42687	1.63097

[$Mn^{3+}{}_2Mn^{4+}{}_2$] eps=38.79

Mn	-0.29122	-1.27868	1.25554
O	-1.38384	-1.40074	-0.37157
Mn	-2.48117	0.02627	0.01711
O	-1.33434	0.37577	1.41305
Mn	-0.19478	1.59748	0.43186
O	-1.34570	1.03329	-1.02193
Mn	-0.30270	-0.47705	-1.72444
O	0.86437	0.21166	0.04976
C	-4.35983	-0.89623	-3.68898
H	-5.01422	-0.01142	-3.73358
H	-3.95517	-1.11591	-4.68562
H	-4.97600	-1.74198	-3.34303
C	-3.24533	-0.65341	-2.69272
O	-3.64453	-0.33309	-1.51394
O	-2.05871	-0.77940	-3.05606
C	-3.83004	3.95419	1.00666
H	-4.69723	3.62927	1.59983

H	-3.30111	4.77340	1.51116
H	-4.20597	4.31576	0.03454
C	-2.90375	2.78708	0.75170
O	-3.47303	1.68196	0.46728
O	-1.66358	2.99461	0.81244
C	-4.33912	-2.62807	2.77617
H	-3.92978	-3.32817	3.51615
H	-4.97675	-1.88169	3.27588
H	-4.97379	-3.17270	2.05855
C	-3.22928	-1.92314	2.02452
O	-2.04107	-2.21513	2.26683
O	-3.63412	-1.05593	1.16693
O	2.18218	-4.12506	-1.11467
V	1.69560	-2.66371	-0.72054
O	0.76590	-2.75102	0.72055
O	0.76005	-2.01744	-2.00717
O	3.15923	-1.63063	-0.44138
V	3.67549	-0.01431	-0.01291
O	5.25639	-0.06325	-0.03135
O	3.24549	1.18512	-1.21319
V	1.70190	1.86632	-1.89650
O	0.78008	0.68741	-2.74761
O	0.76143	2.71725	-0.70675
O	2.16155	2.94893	-2.95974
O	2.18655	1.06865	4.03344
V	1.71944	0.66512	2.57278
O	0.76879	1.99155	1.97449
O	0.80438	-0.78572	2.71707
O	3.25687	0.43142	1.62791

$[Mn^{3+}Mn^{4+}{}_3]$ eps=36.60

Mn	-0.25293	1.52607	-0.69022
Mn	-2.51505	0.03689	0.01348
Mn	-0.36908	-1.51332	-1.05134
Mn	-0.26212	-0.10539	1.66689
O	-1.35989	1.23304	0.84583
O	-1.37885	0.13412	-1.42862
O	0.74572	0.16083	0.11207
O	-1.38699	-1.28277	0.61832
H	-4.06694	-1.39967	4.16342
H	-3.31718	0.01333	4.95978
C	-2.92211	-0.18918	2.85332
C	-3.82514	-0.32909	4.04862
O	-1.67259	-0.24008	3.06103
O	-3.48771	-0.06114	1.71948
H	-4.76254	0.22065	3.88912
H	-5.08202	-2.39282	-2.74777
C	-4.44698	-3.04004	-2.12253
C	-3.31407	-2.22413	-1.55474
O	-2.13171	-2.55264	-1.77641
O	-3.68364	-1.20586	-0.85206
H	-4.06375	-3.88000	-2.71538
H	-5.07202	-3.41426	-1.29622
O	-1.65580	2.78988	-1.31063
O	-3.47888	1.60310	-0.68278
O	0.72859	-1.24568	-2.54917
V	1.68025	0.17406	-2.70761
O	2.10582	0.34597	-4.21830

O	0.75089	1.56264	-2.22458
O	0.73453	-1.53168	2.24620
V	1.66452	-2.47755	1.12071
O	0.71793	-2.82673	-0.26815
O	2.08281	-3.83172	1.81669
O	2.20142	3.39574	2.35628
V	1.72515	2.16209	1.50143
O	0.75458	2.79141	0.19837
O	0.74360	1.16933	2.54415
O	3.22815	1.33166	0.93514
O	5.26179	-0.09380	-0.04574
V	3.68764	-0.05929	-0.02922
O	3.16079	-1.57227	0.67369
O	3.17178	0.07352	-1.69579
H	-3.33158	4.30269	-2.45949
H	-4.78767	3.37504	-1.91353
C	-3.79613	3.75232	-1.63037
C	-2.90633	2.62441	-1.18335
H	-3.91453	4.44382	-0.77853

[$Mn^{3+}Mn^{4+}_3$] eps=36.82

Mn	-0.25294	1.52623	-0.68985
Mn	-2.51506	0.03688	0.01351
Mn	-0.36910	-1.51307	-1.05169
Mn	-0.26212	-0.10579	1.66687
O	-1.35989	1.23285	0.84613
O	-1.37886	0.13447	-1.42857
O	0.74572	0.16080	0.11211
O	-1.38699	-1.28292	0.61803

H	-4.06692	-1.40067	4.16309
H	-3.31715	0.01212	4.95981
C	-2.92209	-0.18985	2.85330
C	-3.82511	-0.33006	4.04857
O	-1.67257	-0.24081	3.06098
O	-3.48769	-0.06155	1.71948
H	-4.76251	0.21972	3.88921
H	-5.08205	-2.39212	-2.74828
C	-4.44697	-3.03952	-2.12326
C	-3.31407	-2.22377	-1.55524
O	-2.13171	-2.55221	-1.77699
O	-3.68364	-1.20566	-0.85231
H	-4.06375	-3.87928	-2.71638
H	-5.07198	-3.41402	-1.29705
O	-1.65580	2.79019	-1.30996
O	-3.47888	1.60326	-0.68237
O	0.72858	-1.24508	-2.54947
V	1.68023	0.17470	-2.70757
O	2.10580	0.34697	-4.21822
O	0.75087	1.56317	-2.22421
O	0.73454	-1.53221	2.24583
V	1.66452	-2.47781	1.12010
O	0.71793	-2.82666	-0.26882
O	2.08282	-3.83215	1.81577
O	2.20143	3.39518	2.35707
V	1.72515	2.16173	1.50193
O	0.75458	2.79136	0.19903
O	0.74362	1.16872	2.54442
O	3.22817	1.33144	0.93544

O	5.26179	-0.09380	-0.04579
V	3.68764	-0.05928	-0.02925
O	3.16079	-1.57243	0.67330
O	3.17177	0.07393	-1.69579
H	-3.33158	4.30331	-2.45841
H	-4.78767	3.37546	-1.91277
C	-3.79615	3.75270	-1.62946
C	-2.90633	2.62469	-1.18271
H	-3.91461	4.44395	-0.77744

[$Mn^{3+}Mn^{4+}_3$] eps=38.79

Mn	-0.25300	1.52622	-0.68986
Mn	-2.51511	0.03687	0.01353
Mn	-0.36913	-1.51306	-1.05166
Mn	-0.26216	-0.10577	1.66686
O	-1.35986	1.23286	0.84614
O	-1.37884	0.13445	-1.42856
O	0.74574	0.16082	0.11210
O	-1.38696	-1.28292	0.61805
H	-4.06684	-1.40068	4.16306
H	-3.31709	0.01211	4.95981
C	-2.92206	-0.18985	2.85331
C	-3.82505	-0.33006	4.04856
O	-1.67251	-0.24077	3.06094
O	-3.48764	-0.06155	1.71947
H	-4.76246	0.21970	3.88922
H	-5.08198	-2.39209	-2.74831
C	-4.44688	-3.03951	-2.12333
C	-3.31403	-2.22377	-1.55528

O	-2.13163	-2.55218	-1.77697
O	-3.68358	-1.20567	-0.85230
H	-4.06362	-3.87922	-2.71650
H	-5.07187	-3.41408	-1.29714
O	-1.65578	2.79013	-1.30996
O	-3.47884	1.60322	-0.68232
O	0.72857	-1.24510	-2.54947
V	1.68019	0.17470	-2.70757
O	2.10581	0.34696	-4.21821
O	0.75084	1.56317	-2.22427
O	0.73457	-1.53219	2.24585
V	1.66452	-2.47778	1.12013
O	0.71793	-2.82667	-0.26878
O	2.08285	-3.83212	1.81580
O	2.20146	3.39517	2.35702
V	1.72514	2.16172	1.50189
O	0.75460	2.79138	0.19901
O	0.74366	1.16873	2.54442
O	3.22822	1.33146	0.93542
O	5.26179	-0.09380	-0.04582
V	3.68764	-0.05927	-0.02925
O	3.16081	-1.57241	0.67331
O	3.17175	0.07394	-1.69578
H	-3.33158	4.30329	-2.45837
H	-4.78765	3.37537	-1.91281
C	-3.79616	3.75263	-1.62946
C	-2.90634	2.62466	-1.18270
H	-3.91468	4.44385	-0.77741

[$Mn^{4+}{}_4$] eps=36.60

Mn	0.32681	-0.44171	1.62722
Mn	2.55568	0.01310	-0.01165
Mn	0.31946	-1.18565	-1.20439
Mn	0.31083	1.63845	-0.43345
O	1.40671	1.02834	1.02231
O	1.41435	-1.39362	0.36108
O	-0.65033	-0.00017	0.00018
O	1.40057	0.39006	-1.40576
H	4.75301	3.59102	-1.51150
H	3.31987	4.70066	-1.55250
C	2.94773	2.75341	-0.72691
C	3.84703	3.92288	-0.98610
O	1.69235	2.95709	-0.78360
O	3.50708	1.64306	-0.44163
H	4.14772	4.33291	-0.00661
H	3.35956	-3.19416	-3.74557
C	3.86491	-2.85992	-2.83059
C	2.96188	-1.98285	-2.01873
O	1.70732	-2.13563	-2.17367
O	3.51543	-1.16716	-1.20954
H	4.80030	-2.33221	-3.06018
H	4.10617	-3.74368	-2.21496
O	1.72083	-0.79323	2.93169
O	3.52288	-0.42662	1.60697
O	-0.68816	-2.63420	-0.75507
V	-1.66380	-2.60220	0.68635
O	-2.06365	-4.06942	1.07131
O	-0.68016	-1.92373	1.95264

O	-0.71017	1.97034	-1.90437
V	-1.68912	0.70310	-2.58765
O	-0.70285	-0.73045	-2.64065
O	-2.10322	1.09889	-4.04802
O	-2.08310	2.95115	2.99041
V	-1.67660	1.88644	1.91246
O	-0.68876	0.66246	2.65899
O	-0.70387	2.65210	0.68838
O	-3.15531	1.16882	1.19915
O	-5.24663	-0.01707	0.01815
V	-3.67955	-0.01155	0.01175
O	-3.16298	0.43146	-1.60524
O	-3.14718	-1.62857	0.43565
H	3.38201	-0.93990	4.81311
H	4.12212	-2.17395	3.74899
C	3.88262	-1.10120	3.84993
C	2.97438	-0.73735	2.71562
H	4.81825	-0.52982	3.78487

[Mn^{4+}_4] eps=36.82

Mn	0.32681	-0.44173	1.62721
Mn	2.55568	0.01310	-0.01165
Mn	0.31946	-1.18563	-1.20440
Mn	0.31083	1.63845	-0.43343
O	1.40671	1.02833	1.02232
O	1.41435	-1.39362	0.36107
O	-0.65033	-0.00017	0.00018
O	1.40057	0.39008	-1.40575
H	4.75297	3.59103	-1.51153

H	3.31985	4.70071	-1.55238
C	2.94773	2.75342	-0.72688
C	3.84703	3.92289	-0.98605
O	1.69235	2.95709	-0.78357
O	3.50707	1.64307	-0.44161
H	4.14779	4.33284	-0.00656
H	3.35955	-3.19416	-3.74558
C	3.86491	-2.85988	-2.83062
C	2.96188	-1.98283	-2.01875
O	1.70732	-2.13560	-2.17370
O	3.51543	-1.16714	-1.20955
H	4.80028	-2.33214	-3.06024
H	4.10621	-3.74362	-2.21498
O	1.72083	-0.79328	2.93168
O	3.52287	-0.42663	1.60696
O	-0.68817	-2.63419	-0.75510
V	-1.66380	-2.60221	0.68632
O	-2.06365	-4.06944	1.07126
O	-0.68017	-1.92375	1.95262
O	-0.71017	1.97036	-1.90435
V	-1.68911	0.70314	-2.58765
O	-0.70285	-0.73042	-2.64066
O	-2.10322	1.09894	-4.04801
O	-2.08310	2.95112	2.99044
V	-1.67660	1.88641	1.91248
O	-0.68876	0.66243	2.65901
O	-0.70387	2.65210	0.68842
O	-3.15531	1.16881	1.19917
O	-5.24663	-0.01707	0.01815

V	-3.67955	-0.01155	0.01174
O	-3.16298	0.43149	-1.60524
O	-3.14719	-1.62858	0.43563
H	3.38198	-0.94007	4.81309
H	4.12226	-2.17394	3.74887
C	3.88262	-1.10123	3.84991
C	2.97438	-0.73739	2.71561
H	4.81820	-0.52975	3.78492

[Mn^{4+}_4] eps=38.79

Mn	0.32695	-0.44171	1.62717
Mn	2.55577	0.01311	-0.01177
Mn	0.31950	-1.18570	-1.20436
Mn	0.31091	1.63845	-0.43353
O	1.40673	1.02838	1.02223
O	1.41443	-1.39361	0.36107
O	-0.65028	-0.00019	0.00014
O	1.40057	0.39002	-1.40584
H	4.75856	3.59099	-1.49980
H	3.32238	4.69587	-1.56208
C	2.94770	2.75347	-0.72701
C	3.84649	3.92342	-0.98555
O	1.69224	2.95704	-0.78380
O	3.50704	1.64315	-0.44172
H	4.13568	4.34139	-0.00592
H	3.35874	-3.19721	-3.74375
C	3.86497	-2.86020	-2.83030
C	2.96188	-1.98300	-2.01869
O	1.70730	-2.13570	-2.17363

O	3.51542	-1.16717	-1.20961
H	4.79905	-2.33127	-3.06250
H	4.10899	-3.74211	-2.21312
O	1.72099	-0.79319	2.93151
O	3.52298	-0.42637	1.60681
O	-0.68819	-2.63433	-0.75506
V	-1.66369	-2.60223	0.68644
O	-2.06350	-4.06944	1.07152
O	-0.67988	-1.92390	1.95261
O	-0.71001	1.97034	-1.90459
V	-1.68906	0.70309	-2.58757
O	-0.70282	-0.73048	-2.64060
O	-2.10342	1.09869	-4.04793
O	-2.08312	2.95115	2.99028
V	-1.67676	1.88637	1.91233
O	-0.68903	0.66228	2.65879
O	-0.70414	2.65198	0.68817
O	-3.15566	1.16899	1.19913
O	-5.24670	-0.01726	0.01797
V	-3.67961	-0.01162	0.01185
O	-3.16286	0.43151	-1.60504
O	-3.14714	-1.62853	0.43601
H	3.38400	-0.93250	4.81311
H	4.11574	-2.17527	3.75378
C	3.88260	-1.10068	3.85002
C	2.97458	-0.73708	2.71550
H	4.82121	-0.53472	3.78132

[($Mn^{3+}Mn^{4+}{}_3$)(H_2O)(OH)] eps=36.82

Mn	0.52785	-1.45479	0.59764
Mn	2.61897	0.01480	-0.58093
Mn	0.09324	0.06083	-1.99318
Mn	0.54026	1.39866	0.70713
O	1.71855	-0.05001	1.07523
O	1.33430	-1.20464	-1.15351
O	-0.58586	-0.00804	0.19187
O	1.35449	1.27244	-1.05477
H	4.32679	4.21481	0.53905
H	3.86967	3.97453	2.24919
C	3.25151	2.42158	0.89078
C	4.23401	3.46760	1.34586
O	2.03068	2.61358	1.17829
O	3.71850	1.44578	0.22479
H	5.22223	3.01977	1.51669
O	2.02740	-2.70868	0.96467
O	3.71112	-1.49171	0.06606
O	-1.11163	-1.30578	-2.45583
V	-1.82647	-2.29284	-1.24944
O	-2.38824	-3.60986	-1.91815
O	-0.62414	-2.72475	-0.07431
O	-0.60388	2.71673	0.11718
V	-1.79962	2.37354	-1.09273
O	-1.08312	1.47425	-2.36810
O	-2.35313	3.73748	-1.66683
O	-1.24143	-0.16165	4.34476
V	-1.07359	-0.10370	2.77900
O	-0.17014	-1.51540	2.30932
O	-0.16309	1.33429	2.41600

O	-2.74833	-0.07008	2.09542
O	-5.08083	-0.00711	0.79814
V	-3.52885	-0.01051	0.52739
O	-3.16956	1.46088	-0.34893
O	-3.19165	-1.42807	-0.43953
H	3.75442	-4.49937	1.32151
H	5.04614	-3.63912	0.38952
C	4.24403	-3.53522	1.13348
C	3.24425	-2.50599	0.68040
H	4.69587	-3.17575	2.07370
H	2.30177	0.19385	-3.29660
O	1.40346	0.26969	-3.74660
H	1.35815	1.19549	-4.04254
O	3.51392	0.05076	-2.20146
H	3.95138	-0.81012	-2.32678

[$(Mn^{3+}Mn^{4+}_3)(H_2O)(OH)$] eps=38.79

Mn	0.52793	-1.45443	0.59836
Mn	2.61910	0.01467	-0.58071
Mn	0.09346	0.06003	-1.99318
Mn	0.54021	1.39900	0.70656
O	1.71841	-0.04943	1.07547
O	1.33435	-1.20506	-1.15279
O	-0.58592	-0.00795	0.19192
O	1.35445	1.27206	-1.05516
H	4.32601	4.21556	0.53785
H	3.86952	3.97523	2.24814
C	3.25134	2.42211	0.89000
C	4.23371	3.46835	1.34472

O	2.03042	2.61417	1.17725
O	3.71837	1.44599	0.22450
H	5.22210	3.02079	1.51526
O	2.02742	-2.70811	0.96596
O	3.71109	-1.49141	0.06694
O	-1.11144	-1.30687	-2.45522
V	-1.82622	-2.29346	-1.24846
O	-2.38792	-3.61080	-1.91664
O	-0.62402	-2.72484	-0.07306
O	-0.60407	2.71679	0.11599
V	-1.79964	2.37298	-1.09387
O	-1.08309	1.47315	-2.36880
O	-2.35322	3.73664	-1.66858
O	-1.24182	-0.15969	4.34477
V	-1.07381	-0.10243	2.77899
O	-0.17032	-1.51433	2.31007
O	-0.16337	1.33542	2.41549
O	-2.74857	-0.06911	2.09531
O	-5.08090	-0.00699	0.79774
V	-3.52888	-0.01042	0.52717
O	-3.16966	1.46056	-0.34984
O	-3.19154	-1.42847	-0.43897
H	3.75377	-4.49719	1.32914
H	5.04174	-3.64393	0.38573
C	4.24422	-3.53492	1.13393
C	3.24429	-2.50564	0.68147
H	4.70262	-3.17185	2.06956
H	2.30192	0.19197	-3.29667
O	1.40355	0.26726	-3.74661

H	1.35843	1.19254	-4.04432
O	3.51396	0.05012	-2.20113
H	3.95236	-0.81037	-2.32597

[($Mn^{4+}{}_4$)(H_2O)(OH)] eps=36.82

Mn	0.53806	-1.47312	0.60486
Mn	2.61820	-0.01166	-0.53434
Mn	0.10132	0.03350	-1.90747
Mn	0.55163	1.44600	0.64803
O	1.73279	-0.02630	1.05313
O	1.36858	-1.23538	-1.12521
O	-0.54133	0.00308	0.00148
O	1.37595	1.25166	-1.09337
H	4.49604	4.07535	0.22684
H	3.84303	4.17722	1.89614
C	3.26654	2.44390	0.76434
C	4.25922	3.50157	1.13900
O	2.05439	2.62457	1.09083
O	3.71724	1.43688	0.11573
H	5.18769	3.03207	1.49386
O	2.01401	-2.68215	1.02642
O	3.69950	-1.47728	0.11536
O	-1.03347	-1.30493	-2.40465
V	-1.81474	-2.29326	-1.20652
O	-2.36440	-3.59371	-1.89178
O	-0.60291	-2.73350	-0.03575
O	-0.57318	2.74140	0.06182
V	-1.81031	2.35149	-1.10419
O	-1.06467	1.38350	-2.32802

O	-2.34836	3.67743	-1.75100
O	-1.19417	-0.06413	4.36242
V	-1.07394	-0.04228	2.79813
O	-0.18018	-1.44363	2.28064
O	-0.16739	1.36686	2.32212
O	-2.72186	-0.03651	2.09069
O	-5.07911	-0.01057	0.81868
V	-3.53648	0.00115	0.53816
O	-3.17455	1.47682	-0.33269
O	-3.16815	-1.42023	-0.42463
H	3.72819	-4.45768	1.44878
H	4.99705	-3.66275	0.42868
C	4.22919	-3.51380	1.20077
C	3.23669	-2.48913	0.74178
H	4.72510	-3.11777	2.10329
O	1.07204	0.03636	-3.46315
H	0.80276	-0.73332	-3.99926
O	3.42564	0.01885	-2.36533
H	2.59917	-0.00114	-2.96761
H	3.88533	0.85881	-2.56131

[($Mn^{4+}{}_4$)(H_2O)(OH)] eps=38.79

Mn	0.53808	-1.47305	0.60493
Mn	2.61827	-0.01166	-0.53431
Mn	0.10137	0.03340	-1.90748
Mn	0.55166	1.44604	0.64794
O	1.73273	-0.02623	1.05316
O	1.36856	-1.23542	-1.12512
O	-0.54136	0.00309	0.00148

O	1.37594	1.25162	-1.09340
H	4.49611	4.07520	0.22664
H	3.84295	4.17740	1.89589
C	3.26652	2.44394	0.76429
C	4.25918	3.50161	1.13889
O	2.05432	2.62462	1.09071
O	3.71719	1.43683	0.11580
H	5.18760	3.03211	1.49390
O	2.01394	-2.68208	1.02655
O	3.69945	-1.47725	0.11549
O	-1.03351	-1.30503	-2.40456
V	-1.81471	-2.29333	-1.20640
O	-2.36438	-3.59382	-1.89160
O	-0.60293	-2.73352	-0.03558
O	-0.57320	2.74144	0.06165
V	-1.81028	2.35142	-1.10434
O	-1.06466	1.38341	-2.32814
O	-2.34841	3.67732	-1.75121
O	-1.19424	-0.06388	4.36241
V	-1.07396	-0.04210	2.79811
O	-0.18023	-1.44348	2.28073
O	-0.16747	1.36702	2.32206
O	-2.72192	-0.03641	2.09068
O	-5.07913	-0.01052	0.81860
V	-3.53648	0.00114	0.53812
O	-3.17454	1.47675	-0.33282
O	-3.16818	-1.42030	-0.42455
H	3.72806	-4.45759	1.44916
H	4.99667	-3.66308	0.42843

C	4.22911	-3.51384	1.20078
C	3.23665	-2.48912	0.74189
H	4.72544	-3.11766	2.10301
O	1.07205	0.03621	-3.46312
H	0.80304	-0.73365	-3.99912
O	3.42574	0.01865	-2.36517
H	2.59940	-0.00117	-2.96756
H	3.88591	0.85835	-2.56125

$[(Mn^{3+}Mn^{4+}{}_3)(H_2O)_2]$ eps=36.82

Mn	0.514922	-1.442602	0.689602
Mn	2.592585	0.010085	-0.468962
Mn	0.097719	-0.011387	-1.939065
Mn	0.505301	1.459289	0.674044
O	1.716431	0.014157	1.111112
O	1.363487	-1.233199	-1.067030
O	-0.585254	0.000707	0.204042
O	1.365635	1.231308	-1.088599
H	4.271977	4.263862	0.323121
H	3.837820	4.130997	2.051301
C	3.221346	2.493232	0.805074
C	4.198116	3.570606	1.178677
O	2.000783	2.685651	1.086536
O	3.691023	1.472124	0.200450
H	5.191864	3.142186	1.364960
O	2.033137	-2.653904	1.108839
O	3.705382	-1.447403	0.179059
O	-1.053317	-1.407463	-2.397218
V	-1.797467	-2.349901	-1.166711

O -2.331979 -3.694154 -1.789014

O -0.605268 -2.722573 0.054218

O -0.623730 2.720299 0.018566

V -1.814255 2.322481 -1.196493

O -1.063745 1.370583 -2.415273

O -2.359756 3.654410 -1.835531

O -1.287888 0.023312 4.336979

V -1.109399 0.013051 2.777171

O -0.180465 -1.414316 2.380685

O -0.192322 1.444749 2.361269

O -2.765676 0.001524 2.073323

O -5.087405 -0.013475 0.749351

V -3.537033 -0.009916 0.495721

O -3.178009 1.430684 -0.433577

O -3.168791 -1.459175 -0.416369

H 3.762683 -4.428897 1.511717

H 5.015652 -3.624375 0.480952

C 4.253812 -3.480614 1.260104

C 3.247400 -2.461114 0.812352

H 4.757544 -3.084808 2.158285

O 1.407952 0.016474 -3.818987

H 1.254516 0.808404 -4.372587

O 3.606690 -0.037908 -2.222743

H 4.099495 -0.874587 -2.334815

H 2.950024 -0.002887 -2.964695

H 1.253400 -0.747931 -4.409494

$[(Mn^{3+}Mn^{4+}{}_3)(H_2O)_2]$ eps=38.79

Mn 0.514932 -1.442471 0.689767

Mn	2.592672	0.010040	-0.468848
Mn	0.097911	-0.011631	-1.939210
Mn	0.505286	1.459351	0.673825
O	1.716326	0.014323	1.111201
O	1.363509	-1.233317	-1.066775
O	-0.585305	0.000737	0.204012
O	1.365637	1.231184	-1.088669
H	4.271919	4.263860	0.322645
H	3.837596	4.131434	2.050830
C	3.221247	2.493388	0.804918
C	4.197966	3.570828	1.178376
O	2.000632	2.685802	1.086279
O	3.690945	1.472175	0.200497
H	5.191691	3.142438	1.364851
O	2.033021	-2.653754	1.109209
O	3.705315	-1.447279	0.179514
O	-1.053275	-1.407745	-2.397043
V	-1.797405	-2.350021	-1.166456
O	-2.331964	-3.694346	-1.788593
O	-0.605299	-2.722572	0.054566
O	-0.623782	2.720324	0.018185
V	-1.814213	2.322288	-1.196860
O	-1.063711	1.370241	-2.415478
O	-2.359774	3.654118	-1.836087
O	-1.288068	0.023875	4.336944
V	-1.109494	0.013409	2.777126
O	-0.180593	-1.414000	2.380889
O	-0.192487	1.445064	2.361090
O	-2.765807	0.001775	2.073268

O -5.087461 -0.013387 0.749192

V -3.537058 -0.009859 0.495625

O -3.178030 1.430626 -0.433814

O -3.168809 -1.459232 -0.416237

H 3.762457 -4.428634 1.512708

H 5.014854 -3.625023 0.480561

C 4.253695 -3.480630 1.260271

C 3.247323 -2.461036 0.812722

H 4.758309 -3.084510 2.157815

O 1.408303 0.015675 -3.818725

H 1.255192 0.807207 -4.373013

O 3.606862 -0.037962 -2.222374

H 4.100046 -0.874438 -2.334501

H 2.950342 -0.003073 -2.964481

H 1.254595 -0.749176 -4.408909

$[(Mn^{4+}{}_4)(H_2O)_2]$ eps=36.82

Mn 0.546399 -1.513387 0.584374

Mn 2.619133 -0.006909 -0.529491

Mn 0.044910 0.050905 -1.811707

Mn 0.565679 1.460724 0.670348

O 1.720805 -0.045306 1.036293

O 1.368391 -1.214416 -1.170398

O -0.547853 0.001604 -0.034688

O 1.375534 1.258808 -1.091487

H 4.687999 3.912167 0.365126

H 3.790329 4.202246 1.901764

C 3.261869 2.420118 0.826831

C 4.259823 3.442631 1.265874

O	2.041607	2.606258	1.137158
O	3.705082	1.425085	0.158560
H	5.077376	2.934734	1.800042
O	1.994640	-2.704318	0.989678
O	3.683160	-1.484237	0.107198
O	-1.052565	-1.265240	-2.383411
V	-1.841525	-2.295497	-1.207568
O	-2.388289	-3.576897	-1.918475
O	-0.600605	-2.748488	-0.067790
O	-0.563979	2.749963	0.095395
V	-1.811104	2.381546	-1.067557
O	-1.035870	1.411496	-2.302827
O	-2.341649	3.709238	-1.701744
O	-1.173273	-0.123239	4.361100
V	-1.071850	-0.077919	2.800675
O	-0.181311	-1.471458	2.236941
O	-0.161921	1.332541	2.320930
O	-2.712088	-0.047444	2.091458
O	-5.081141	0.005913	0.834867
V	-3.544180	0.004171	0.548863
O	-3.158389	1.482628	-0.320965
O	-3.177297	-1.423866	-0.409269
H	3.702104	-4.484471	1.392762
H	4.987702	-3.667696	0.408216
C	4.207047	-3.536438	1.170484
C	3.224229	-2.505360	0.716212
H	4.684915	-3.154217	2.088881
O	1.023165	0.085079	-3.614641
H	0.811970	0.892276	-4.133863

O 3.576985 0.057339 -2.301306

H 2.929737 0.019962 -3.040055

H 4.118975 0.859973 -2.449421

H 0.762133 -0.675322 -4.180096

[($Mn^{4+}{}_4$)(H_2O)$_2$] eps=38.79

Mn 0.546388 -1.513370 0.584304

Mn 2.619208 -0.006899 -0.529393

Mn 0.045032 0.050971 -1.811787

Mn 0.565681 1.460664 0.670391

O 1.720717 -0.045355 1.036377

O 1.368406 -1.214395 -1.170342

O -0.547873 0.001599 -0.034742

O 1.375550 1.258839 -1.091345

H 4.686879 3.912889 0.364760

H 3.790327 4.202014 1.902206

C 3.261809 2.420116 0.826956

C 4.259666 3.442820 1.265698

O 2.041511 2.606204 1.137288

O 3.705041 1.424987 0.158854

H 5.077873 2.935080 1.799000

O 1.994533 -2.704343 0.989683

O 3.683111 -1.484247 0.107352

O -1.052515 -1.265155 -2.383507

V -1.841464 -2.295423 -1.207732

O -2.388267 -3.576807 -1.918674

O -0.600654 -2.748497 -0.067903

O -0.564030 2.749962 0.095498

V -1.811054 2.381560 -1.067526

O	-1.035854	1.411564	-2.302794
O	-2.341639	3.709280	-1.701661
O	-1.173457	-0.123401	4.361079
V	-1.071957	-0.078013	2.800635
O	-0.181476	-1.471535	2.236897
O	-0.162058	1.332455	2.321002
O	-2.712228	-0.047517	2.091403
O	-5.081199	0.005869	0.834691
V	-3.544199	0.004153	0.548752
O	-3.158420	1.482652	-0.320952
O	-3.177307	-1.423833	-0.409414
H	3.701920	-4.484395	1.393289
H	4.987165	-3.668230	0.407794
C	4.206947	-3.536544	1.170443
C	3.224161	-2.505414	0.716272
H	4.685405	-3.154068	2.088425
O	1.023491	0.085188	-3.614344
H	0.813179	0.892646	-4.133552
O	3.577283	0.057361	-2.300908
H	2.930390	0.019595	-3.039954
H	4.119213	0.860052	-2.449102
H	0.762580	-0.674959	-4.180225
