# Supplementary material for: Activation by oxidation and ligand exchange in a molecular manganese vanadium oxide water oxidation catalyst
Source: Chem Sci. 2021 Aug 30;12(39):12918–27. doi: 10.1039/d1sc03239a (PMC8513927; doi:10.1039/d1sc03239a)
Supplement: SC-012-D1SC03239A-s002 [file SC-012-D1SC03239A-s002.pdf]

Electronic Supplementary Material (ESI) for Chemical Science.
This journal is © The Royal Society of Chemistry 2021

Supporting Information

Activation by oxidation and ligand exchange in a molecular manganese vanadium oxide water oxidation catalyst

Gustavo Cardenas,[a,b] Ivan Trentin,[c] Ludwig Schwiedrzik,[a] David Hernández-Castillo,[a] Grace A. Lowe,[c] Julian Kund,[d] Christine Kranz,[d] Sarah Klingler,[d] Robert Stach[d] Boris Mizaikoff,[d] Philipp Marquetand,[a,e] Juan J. Nogueira,[b,e] Carsten Streb,*[c] and Leticia González*[a,f]

a. Institute of Theoretical Chemistry, University of Vienna, Währinger Str. 17, 1090 Vienna, Austria.

b. Chemistry Department, Universidad Autónoma de Madrid, Calle Francisco Tomás y Valiente, 7, 28049 Madrid, Spain.

c. Institute of Inorganic Chemistry I, Ulm University, Albert-Einstein-Allee 11, 89081, Ulm, Germany.

d. Institute of Analytical and Bioanalytical Chemistry, Ulm University, Albert-Einstein-Allee 11, 89081 Ulm, Germany

e. IADCHEM, Institute for Advanced Research in Chemistry, Universidad Autónoma de Madrid, Madrid, Spain

f. Vienna Research Platform on Accelerating Reaction Discovery, University of Vienna Währinger Str. 17, 1090 Vienna, Austria

Table of Contents

S1. Electrochemistry

S2. Calculation of Ligand Exchange Pathways

S3. Calculation of Redox Potentials

S1. Electrochemistry

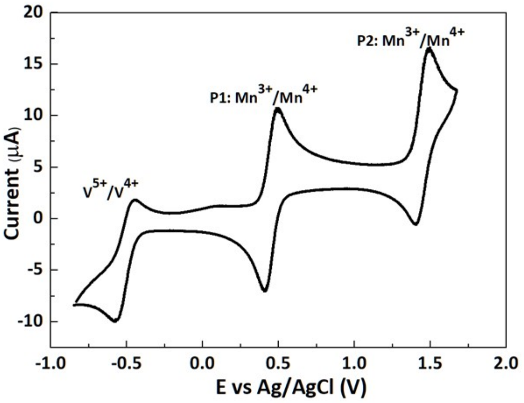

Figure S1. Exemplary cyclovoltammogram of the native cluster {Mn_4V_4} (2 mM, oxidation state [$Mn^{3+}{}_2Mn^{4+}{}_2$]) in water-free MeCN solution (containing 0.1 M $n$$Bu_4NPF_6$ as electrolyte) recorded at a glassy carbon electrode (3 mm diam.) scan rate: 50 mV/s vs. Ag/AgCl.

Square wave voltammetry

While cyclic voltammetry (CV) is a standard method to evaluate electron transfer kinetic data and reaction mechanisms in electrochemical experiments, square-wave voltammetry (SWV) offers significant advantages for the system studied here. In contrast to CV, where capacitive current may mask the faradaic signal for redox active species evolving at low concentration, SWV eliminates the capacitive current component as it is a differential method and thus achieves significantly higher sensitivity (up to at least 3 magnitudes, also see Figure S2).[1] In addition, recording the forward and backward scan and varying either the scan frequency *f* or the current amplitude E_{SW} allows the effective study of electron transfer kinetics and reaction mechanisms.[2] Depending on the experimental conditions, SWV can differentiate two or more closely spaced redox processes by providing a better peak separation (resolution) in comparison to CV. Hence, SWV was chosen here as electrochemical method to study the electrochemistry of $[Mn_4V_4O_{17}(OAc)_3]^{3-}$ (abbreviated hereafter as **{Mn_4V_4}**).

As shown in Figure S2, when comparing CV and SWV data for the first two oxidation redox processes (P1, P2) at different water concentrations, marked differences are observed. While the CV data is dominated by capacitive currents, the SWV data is more sensitive and allows observations of five distinguishable redox processes. Based on the CV data, the quasi-reversibility of P1 and P2 are observed. In addition, we note that the peak separation ΔE_p increases with increasing water content, see Table S1.

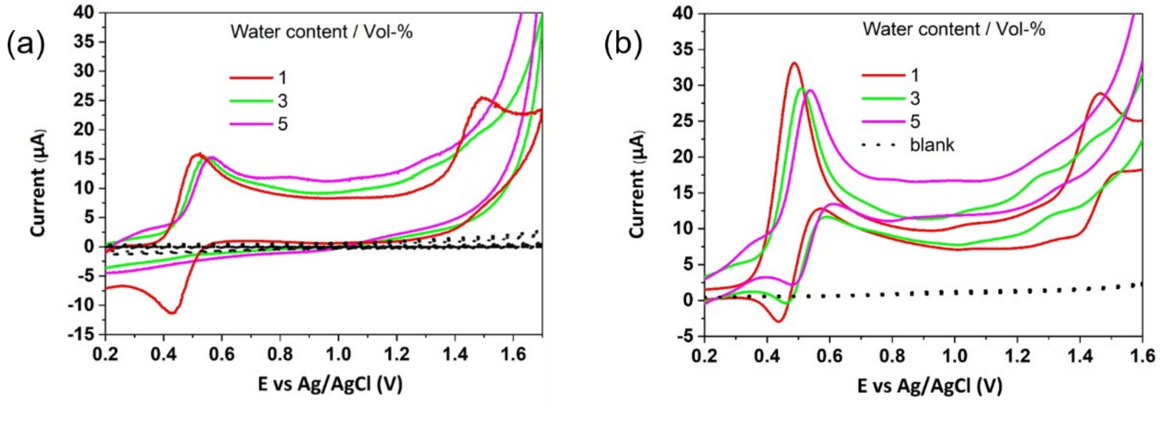

Figure S2: (a) CV of **{Mn_4V_4}** at 1, 3, 5 vol-% water content, scan rate = 50 mV/s; (b) SWV of **{Mn_4V_4}** at 1, 3, 5 vol-% water content *f*= 25 Hz, E_{SW} = 25 mV, E_{step}= 2 mV. Conditions: solvent: MeCN with added demineralized water and 0.1 M nBu_4NPF_6; [**{Mn_4V_4}**] = 2 Mm.

Table S1: CV peak separation depending on water content of the solvent

Water content / vol-%	$\Delta E_{p,}$ P1 / mV	$\Delta E_{p,}$ P2 / mV
0	73	90
1	84	>100[a]
2	>100[a]	-[b]

[a]reductive peak shows low S/N ratio; [b]reductive peak not observed

SWV analysis of the individual oxidation and ligand exchange processes

For the non-oxidized, native catalyst [$Mn^{3+}{}_2Mn^{4+}{}_2$], no acetate-to-water water ligand exchange is observed on the timescale of SWV experiments, as shown by time-dependent ATR-IR spectroscopy (Figure 2a in the ms). However, for the one-electron oxidized species [$Mn^{3+}Mn^{4+}{}_3$] electrochemical data and theory suggest that this ligand exchange is significantly faster. This is in line with SWV data, where the current of the second oxidation step P2 reduces significantly with increasing water content, while a new redox active species, P4, appears at slightly less positive potentials than P2. Theoretical data suggests that P4 could be due to the oxidation of the ligand-exchanged [$Mn^{3+}Mn^{4+}{}_3$], see Figure S3. In sum, these observations and calculations suggest that two independent oxidation paths, *i.e.* P2 (2nd oxidation step without ligand exchange) and P4 (ligand exchanged species that undergoes a second oxidation), are accessible starting from the non-ligand exchanged species [$Mn^{3+}Mn^{4+}{}_3$], see Figure S3.

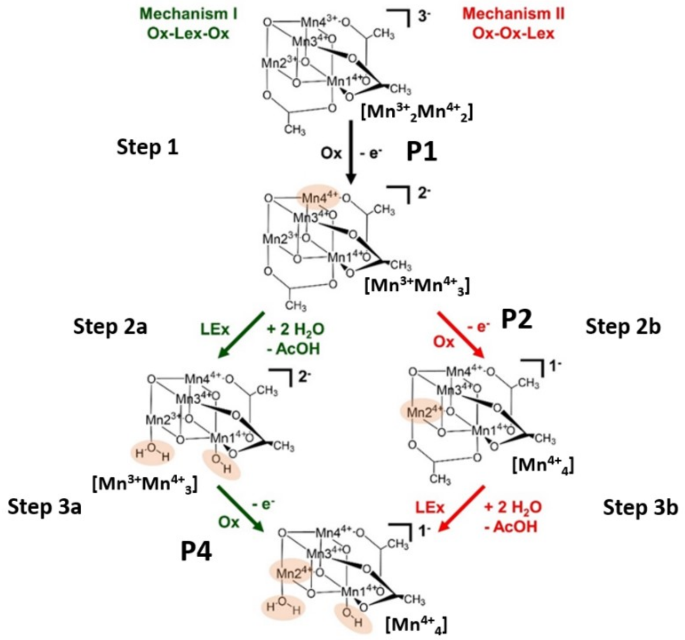

Figure S3: Proposed oxidation (Ox) and ligand exchange (LEx) mechanisms together with oxidation peak assignments P1, P2, P4. Also see discussion in main text.

The correlation of the peaks P2 and P4 was further investigated by variation of the SWV frequency. This method (in analogy to scan rate variation in CV) allows frequency-dependent analyses of electron transfer processes and their kinetics, while retaining the advantages of SWV over CV. Here, we used frequency-variation SWV to gain further insights into the observed electrochemical processes P1 – P5. Variation of the SWV frequency between 5 and 50 Hz showed that with increasing frequency (analogous to faster scan rate in CV), process P2 is partially recovered (Figure S4a,b). This is in line with the interpretation that at higher frequency, the [$Mn^{3+}Mn^{4+}{}_3$] → [$Mn^{4+}{}_4$] oxidation is preferred, as the rate constant for ligand exchange is lower than the electron transfer rate constant for

this oxidation step. (Figure S3, Step 2a). Thus, at high frequency, the oxidation can compete with the acetate-to-water ligand exchange, see Figure S3, Step 2b.

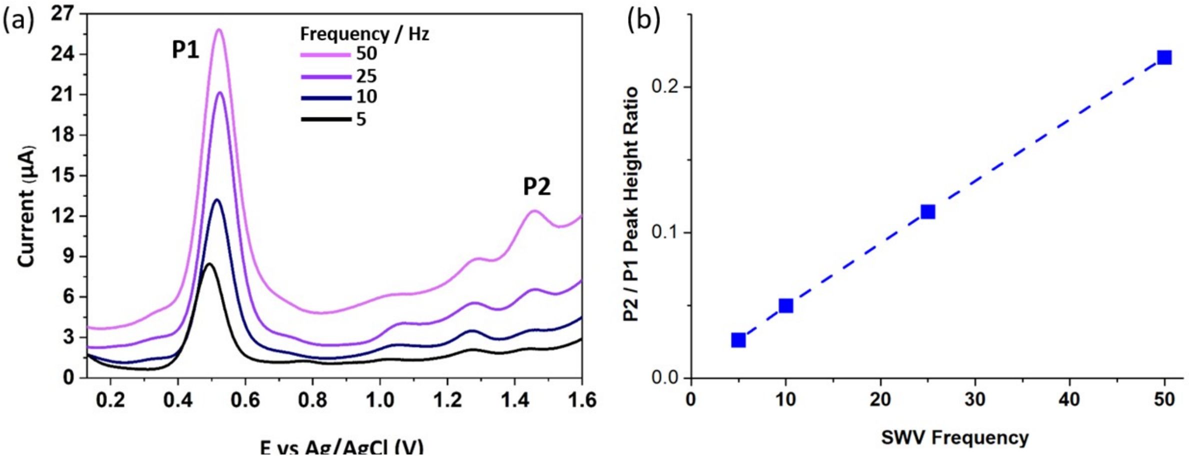

Figure S4: (a) Frequency-dependent SWV, with frequency variation from 5 Hz to 50 Hz, E_{SW} = 25 mV, E_{step}= 2 mV, [**{Mn_4V_4}**] = 2 mM, solvent: acetonitrile containing 3 vol-% water and 0.1 M *n*Bu_4NPF_6. (b) Ratio of P2 / P1 peak heights, showing the partial recovery of peak P2 at increasing SWV frequencies.

Analysis of 10 consecutive SWV scans shows decreasing currents which are indicative of surface deposition of {Mn_4V_4}-related species, as shown in Figure S5.

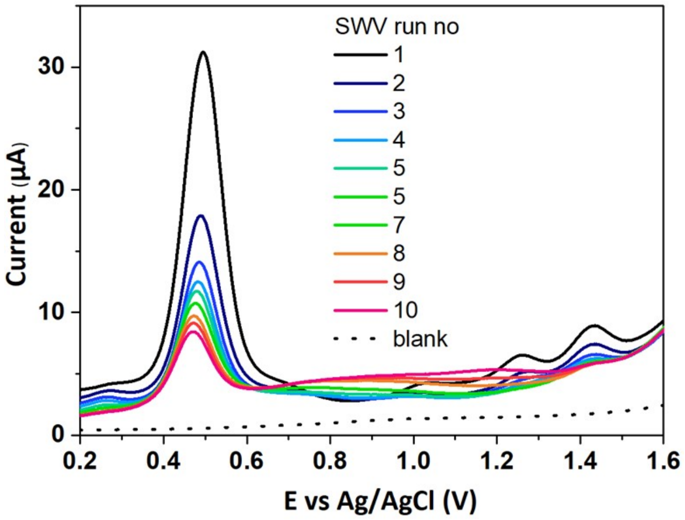

Figure S5: Ten consecutive SWV sweeps. Conditions: [**{Mn_4V_4}**] = 2 mM, *f* = 25 Hz, E_{SW} = 25 mV, E_{step}= 2 mV, solvent: acetonitrile containing 3 vol-% water and 0.1 M *n*Bu_4NPF_6.

S2. Calculation of ligand exchange pathways

In pathways Ia-Ic (Ox-LEx-Ox mechanism), the presence of a Mn^{3+} ion in an octahedral environment gives rise to Jahn-Teller (JT) distortions, elongating one bond axis (the JT axis) and shortening the other two. Due to the different positions where the Mn^{3+} ion could be located, but also due to the symmetry equivalence of the Mn2, Mn3 and Mn4 atoms (in the C_{3v} group), there are six possible orientations of the JT axis in the [$Mn^{3+}Mn^{4+}{}_3$] reactant species (Figure S6). For this reason, geometry optimizations were carried out starting from six guess structures, where elongated Mn-O bonds corresponding to each possible orientation of the JT axis were deliberately constrained. Upon releasing the constraints, all optimizations converged toward one structure, where the Mn^{3+} ion is Mn2, with the JT axis along the Mn-acetate bond (Figure S6, e).

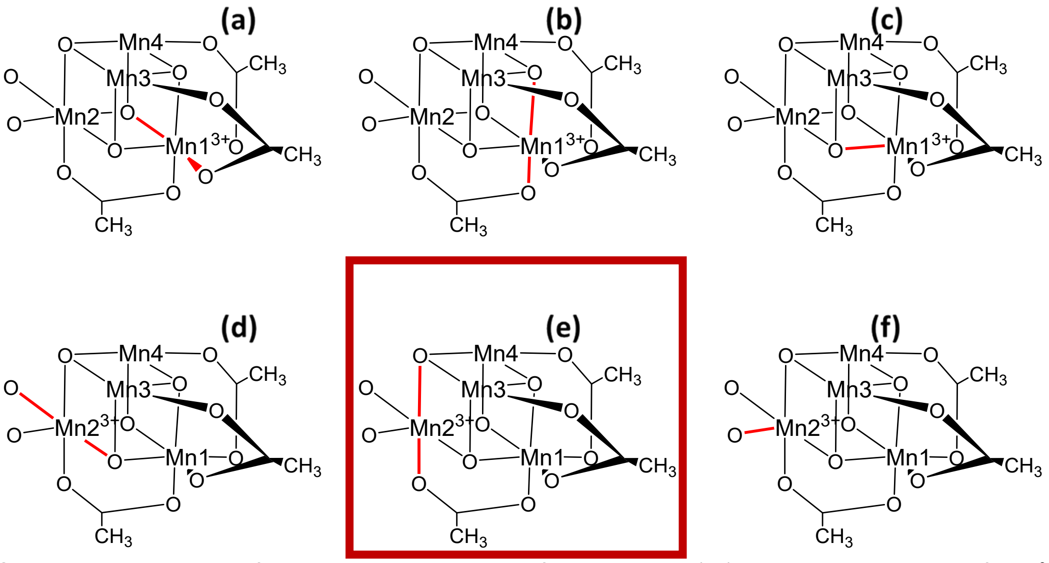

Figure S6. Schematic representation of the six possible orientations of the Jahn-Teller (JT) axis due to the presence of a Mn^{3+} ion. The geometry optimizations of the guess structures (Mn-O bonds deliberately elongated to constrain the localization of the JT axis are shown in red) converged in all cases towards the structure represented by (e).

All pathways Ia-Ic and IId-IIe were determined stepwise, by first optimizing the transition state (TS) of a ligand exchange reaction, followed by the optimization of the two intermediates connected by the TS using the two extrema of the oscillation associated with the imaginary frequency as guess structures for the reactant and the product. This process was repeated for the structures involved in the second ligand exchange reaction of each pathway. More specifically, for the TS determination we first performed a constrained geometry optimization, in which the atoms participating in the ligand substitution were frozen, followed by a relaxed TS optimization. The Gibbs free energies of all structures considered are collected in Table S2 and depicted in Figure 4 of the main manuscript (pathways Ia-Ic and IId), and Figure S7 (pathway IIe). Cartesian coordinates of all geometries are provided separately (file coordinates.pdf).

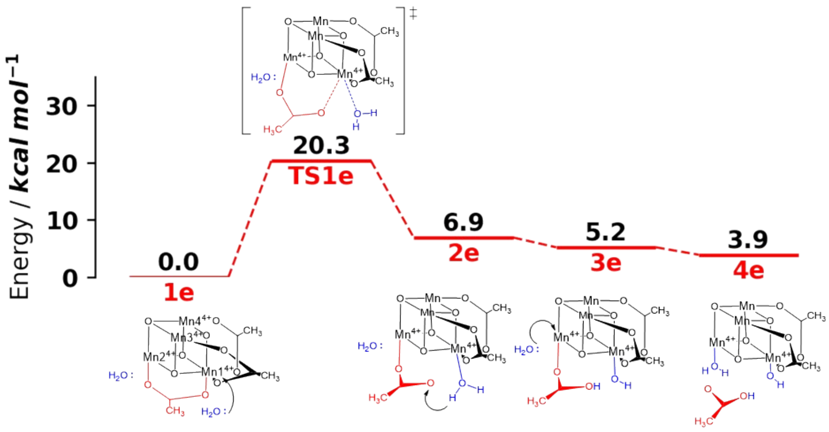

Figure S7. Energy diagram (ΔG free energy in kcal/mol) of the ligand exchange pathway IIe (Ox-Ox-LEx) studied at the B3LYP-D3/Def2-TZVP(Mn,V,O)-Def2-SVP(C,H)//B3LYP-D3/Def2-SVP level of theory. Oxidation numbers are only shown at the cubane center when they change. The structures below and above the energy diagram are reaction intermediates and transition states, respectively. Energies relative to the reactant species. Note that a species with a OH group and a H_2O molecule as ligands is formed.

Localization of the Mn^{3+} and Mn^{4+} ions is monitored along all pathways by inspecting Mulliken spin populations (Tables S3 and S4). Spin populations close to the ideal values of 4 and 3 correspond to Mn^{3+} and Mn^{4+} ions, respectively, so that electron transfer processes can be easily shown, *e.g,* as in **2a→3a**. These electron transfer processes can also be evinced structurally, by observing the Mn ion that presents a JT distortion characteristic of a Mn^{3+} ion. On the one hand, in **2a** the Mn2-OH_2 bond length is 2.17 Å and the parallel Mn2-O_{cub} bond (with a cubane oxygen atom O_{cub}) length amounts to 2.30 Å, whereas the Mn1-OAc bond is 1.90 Å, and the parallel Mn1-O_{cub} bond length amounts to 1.85 Å. On the other hand, for **3a** the analogous bonds involving Mn2 present distances of 1.96 Å (Mn2-OH_2) and 1.92 Å (Mn2-O_{cub}), whereas bonds involving Mn1 have bond lengths of 2.06 Å (Mn1-OH_2) and of 2.15 Å (Mn1-O_{cub}). The latter clearly evidences the JT axis displacement concomitant with the electron transfer process from Mn2 to Mn1 (see table S5 for a comprehensive list of all Mn-O bond lengths of the bonds involved in the ligand substitution reactions).

Table S2. Gibbs Free Energies (in kcal/mol) of all ligand exchange pathways studied in the present work.

Path Ia		Path Ib		Path Ic		Path IId		Path IIe	
Structure	ΔG (kcal/mol)	Structure	ΔG (kcal/mol)	Structure	ΔG (kcal/mol)	Structure	ΔG (kcal/mol)	Structure	ΔG (kcal/mol)
1a	0.0	1b	0.0	1c	0.0	1d	0.0	1e	0.0
TS1a	7.3	TS1b	7.3	TS1c	21.0	TS1d	20.4	TS1e	20.3
2a	1.6	2b	1.6	2c	0.9	2d	6.8	2e	6.9
3a	8.7	TS2b	28.3	TS2c	34.0	3d	6.2	3e	5.2
4a	7.3	3b	0.3	3c	6.8	TS2d	26.0	4e	3.9
TS2a	15.3					4d	4.3		
5a	8.5								
6a	0.3								

Table S3. Mulliken spin populations of the Mn ions of all structures involved in pathways in which the ligand exchange reactions occur before the oxidation reaction (Ox-LEx-OX).

Pathway	Structure	Mn1	Mn2	Mn3	Mn4
Ia and Ib	1a/1b	2.98	3.87	3.04	2.98
	TS1a/TS1b	2.97	3.90	3.05	2.98
	2a/2b	3.02	3.89	3.05	3.02
	TS2b	2.97	3.87	3.18	2.97
	3a	3.89	2.99	2.99	2.98
	4a	3.90	3.00	2.99	2.99
	TS2a	3.92	2.99	2.98	2.99
	5a	3.91	3.00	2.98	3.00
	6a	3.00	3.88	3.01	2.99
Ic	1c	3.01	3.87	3.06	3.01
	TS1c	3.14	3.88	3.06	3.02
	2c	3.03	3.88	3.06	3.01
	TS2c	3.03	3.86	3.16	2.97
	3c	3.03	3.88	3.08	3.02

Table S4. Mulliken spin populations of the Mn ions of all structures involved in pathways in which the ligand exchange reactions occur after the oxidation reaction (Ox-Ox-LEx).

Pathway	Structure	Mn1	Mn2	Mn3	Mn4
IId	1d/1e	3.06	2.95	2.93	2.93
	TS1d	3.03	3.05	2.92	2.92
	2d	3.03	2.96	2.95	2.94
	3d	3.07	2.95	2.95	2.95
	TS2d	3.17	2.96	2.94	2.93
	4d	3.07	2.96	2.95	2.95
IIe	TS1e	3.16	2.97	2.93	2.94
	2e	3.07	2.94	2.95	2.94
	3e	3.03	2.96	2.95	2.95
	4e	3.03	2.93	2.94	2.92

Table S5. Bond lengths of the Mn-O bonds involved in the ligand exchange reactions of all the intermediates involved in the pathways studied in this work. The bond lengths of the bonds breaking/formatting on the TS structures are not indicated, and are simply referred to as "TS bonds".

		Mn (VO-bound)		Mn (apex)	
Pathway	Species	Bond	Bond Length (Å)	Bond	Bond Length (Å)
Ia	1a/b	Mn2-OAc	2.19	Mn1-OAc	1.91
		Mn2-O_{cub}	2.31	Mn1-O_{cub}	1.86
	TS1a/b	Mn2 (TS bonds)		Mn1-OAc	1.89
		Mn2-O_{cub}	2.29	Mn1-O_{cub}	1.85
	2a/b	Mn2-OH	2.17	Mn1-OAc	1.90
		Mn2-O_{cub}	2.30	Mn1-O_{cub}	1.85
	3a/b	Mn2-OH	1.96	Mn1-OAc	2.06
		Mn2-O_{cub}	1.91	Mn1-O_{cub}	2.15
	4a	Mn2-OH	1.85	Mn1-OAc	2.13
		Mn2-O_{cub}	1.98	Mn1-O_{cub}	2.12
	TS2a	Mn2-OH	1.84	Mn1 (TS bonds)	
		Mn2-O_{cub}	2.00	Mn1-O_{cub}	2.12
	5a	Mn2-OH	1.84	Mn1-OH_2	2.18
		Mn2-O_{cub}	2.00	Mn1-O_{cub}	2.15
	6a	Mn2-OH_2	2.18	Mn1-OH	1.85
		Mn2-O_{cub}	2.30	Mn1-O_{cub}	1.88
Ib	TS2b	Mn2-OH_2	2.15	Mn1-O_{cub}	1.85
		Mn2-O_{cub}	2.32	Mn1 (TS bonds)	
Ic	1c	Mn3-OAc	2.00	Mn1-OAc	1.96
		Mn3-O_{cub}	1.87	Mn1-O_{cub}	1.84
	TS1c	Mn3 (TS bonds)		Mn1-OAc	1.90
		Mn3-O_{cub}	1.84	Mn1-O_{cub}	1.86
	2c	Mn3-OH_2	2.00	Mn1-OAc	1.92
		Mn3-O_{cub}	1.85	Mn1-O_{cub}	1.83
	TS2c	Mn3-OH_2	1.96	Mn1-O_{cub}	1.80
		Mn3-O_{cub}	1.87	Mn1 (TS bonds)	
	3c	Mn3-OH	1.91	Mn1-OH_2	2.00
		Mn3-O_{cub}	1.90	Mn1-O_{cub}	1.82

S3. Calculation of Redox Potentials

The redox processes considered were (also see step-labelling in Figure S3):

I. **Step 1**: First oxidation of the manganese vanadium oxide: $[Mn^{3+}{}_2Mn^{4+}{}_2]$→$[Mn^{3+}Mn^{4+}{}_3]$ + e^-
II. **Step 2b:** Second oxidation of the manganese vanadium oxide: $[Mn^{3+}Mn^{4+}{}_3]$→$[Mn^{4+}{}_4]$ + e^-
III. **Step 3a:** Second oxidation of the manganese vanadium oxide after ligand exchange and deprotonation (Step 2a): $[(Mn^{3+}Mn^{4+}{}_3)(H_2O)(OH)]$→$[(Mn^{4+}{}_4)(H_2O)(OH)]$ + e^-
IV. Second oxidation of the manganese vanadium oxide after ligand exchange only (for comparison): $[(Mn^{3+}Mn^{4+}{}_3)(H_2O)_2]$→$[(Mn^{4+}{}_4)(H_2O)_2]$ + e^-

For the species $[Mn^{3+}Mn^{4+}{}_3]$, $[Mn^{4+}{}_4]$, $[(Mn^{3+}Mn^{4+}{}_3)(H_2O)(OH)]$ and $[(Mn^{4+}{}_4)(H_2O)(OH)]$, initial guesses were taken from the ligand exchange pathway studies. In the native manganese vanadium oxide $[Mn^{3+}{}_2Mn^{4+}{}_2]$, it was found that the JT distortion is located along the Mn-acetate bond of two symmetry equivalent Mn^{3+}, see Figure S8. Thus, this configuration was used as an initial guess structure for the calculations.

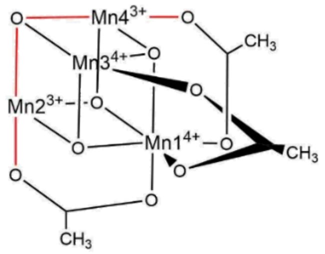

Figure S8. Schematic representation of the JT axes in the most stable configuration found for the $[Mn_3{}^{+2}Mn_4{}^{+2}]$ species.

The standard reduction potentials $\Delta E^{\circ}_{reduction}$ resulting from our calculations are in Table S6. No calculations were carried out at 0% water for the $[(Mn^{3+}Mn^{4+}{}_3)(H_2O)(OH)]$→$[(Mn^{4+}{}_4)(H_2O)(OH)]$ + e^- reaction involving the manganese vanadium oxide with an H_2O and an OH ligand as well as the $[(Mn^{3+}Mn^{4+}{}_3)(H_2O)_2]$→$[(Mn^{4+}{}_4)(H_2O)_2]$ + e^- reaction involving the manganese vanadium oxide with two H_2O ligands, as these species can only be formed in the presence of water. The significantly higher reduction potential of the reaction involving the species with two H_2O ligands (1.62 V on average) compared to that of the species involving an H_2O and an OH ligand that was predicted by our pathway calculations (1.22 V on average) further supports the conclusion that ligand exchange is most likely followed by immediate deprotonation, as seen in pathway Ia.

Table S6. Calculated standard reduction potentials $\Delta E^{\circ}_{reduction}$ for all redox processes and solvent compositions discussed in this section.

Reaction	0% water	0,5% water	5%water
$[Mn^{3+}{}_2Mn^{4+}{}_2]$→$[Mn^{3+}Mn^{4+}{}_3]$ + e^-	0.37	0.37	0.39
$[Mn^{3+}Mn^{4+}{}_3]$→$[Mn^{4+}{}_4]$ + e^-	1.34	1.34	1.34
$[(Mn^{3+}Mn^{4+}{}_3)(H_2O)(OH)]$→$[(Mn^{4+}{}_4)(H_2O)(OH)]$ + e^-	-	1.21	1.22
$[(Mn^{3+}Mn^{4+}{}_3)(H_2O)_2]$→$[(Mn^{4+}{}_4)(H_2O)_2]$ + e^-	-	1.62	1.62

Literature

1 A. Chen and B. Shah, *Anal. Methods*, 2013, **5**, 2158.
2 S. N. Vettorelo and F. Garay, *J. Solid State Electrochem.*, 2016, **20**, 3271–3278.
